# Travel scenario workshops for geographical accessibility modeling of health services: A transdisciplinary evaluation study

**DOI:** 10.3389/fpubh.2022.1051522

**Published:** 2023-01-18

**Authors:** Lotte Molenaar, Fleur Hierink, Michel Brun, Jean-Pierre Monet, Nicolas Ray

**Affiliations:** ^1^Faculty of Medicine, Institute of Global Health, University of Geneva, Geneva, Switzerland; ^2^GeoHealth Group, Institute for Environmental Sciences, University of Geneva, Geneva, Switzerland; ^3^Faculty of Science, Athena Institute, Vrije Universiteit Amsterdam, Amsterdam, Netherlands; ^4^Technical Division, United Nations Population Fund (UNFPA), New York, NY, United States

**Keywords:** geographic accessibility, maternal health, emergency obstetric care, travel scenario, interactive learning

## Abstract

**Introduction:**

Limited geographical access to quality Emergency Obstetric and Newborn Care (EmONC) is a major driver of high maternal mortality. Geographic access to EmONC facilities is identified by the global community as a critical issue for reducing maternal mortality and is proposed as a global indicator by the Ending Preventable Maternal Mortality (EPMM) initiative. Geographic accessibility models can provide insight into the population that lacks adequate access and on the optimal distribution of facilities and resources. Travel scenarios (i.e., modes and speed of transport) used to compute geographical access to healthcare are a key input to these models and should approximate reality as much as possible. This study explores strategies to optimize and harmonize knowledge elicitation practices for developing travel scenarios.

**Methods:**

Knowledge elicitation practices for travel scenario workshops (TSW) were studied in 14 African and South-Asian countries where the United Nations Population Fund supported ministries of health and governments in strengthening networks of EmONC facilities. This was done through a mixed methods evaluation study following a transdisciplinary approach, applying the four phases of the Interactive Learning and Action methodology: exploration, in-depth, integration, and prioritization and action planning. Data was collected in November 2020–June 2021 and involved scoping activities, stakeholder identification, semi-structured interviews (*N* = 9), an evaluation survey (*N* = 31), and two co-creating focus group discussions (*N* = 8).

**Results:**

Estimating realistic travel speeds and limited time for the workshop were considered as the largest barriers. The identified opportunities were inclusively prioritized, whereby preparation; a favorable composition of attendees; validation practices; and evaluation were anticipated to be the most promising improvement strategies, explaining their central place on the co-developed initial standard operating procedure (SOP) for future TSWs. Mostly extensive preparation—both on the side of the organization and the attendees—was anticipated to address nearly all of the identified TSW challenges.

**Conclusion:**

This study showed that the different identified stakeholders had contradicting, complementing and overlapping ideas about strategies to optimize and harmonize TSWs. Yet, an initial SOP was inclusively developed, emphasizing practices for before, during and after each TSW. This SOP is not only relevant in the context of the UNFPA EmONC development approach, but also for monitoring the newly launched EPMM indicator and even in the broader field of geographic accessibility modeling.

## 1. Introduction

Accessible and quality emergency obstetric and newborn care (EmONC) are essential to prevent the main causes of maternal mortality, namely hemorrhage, hypertensive disorders and sepsis (1–5). While globally the maternal mortality ratio (MMR) has been steadily decreasing over the past two decades, large discrepancies exist across the regions of the world, with low income countries still bearing a MMR of 462 deaths per 100,000 live births in 2017—compared to 11 deaths in 100,000 births in high income countries—and with Sub-Saharan Africa and Southern Asia accounting for 86% of all maternal deaths (1, 5, 6). Timely and adequate access to EmONC services has been identified as one of the key targets to further reduce preventable maternal deaths in all regions of the world. A recently published global target of the World Health Organization (WHO)/United Nations Population Fund (UNFPA) *Ending Preventable Maternal Mortality* (EPMM) initiative states that by 2025, at least 60% of the population should be covered by *functional* EmONC facilities within 2 h travel time (7, 8). Here, the EPMM indicator refers to the geographical accessibility, which indicates how easily (pregnant) women can physically access an EmONC facility within a given travel time (7, 9). Geographical accessibility models shine light on the portion of the population that has access to certain health services by taking into account physical barriers and facilitators of movement—such as mountains and the status of transportation networks (10). The results of geographic accessibility models focusing on travel time can support the optimal distribution of EmONC services and help monitor the recently launched global EPMM target by tracking population coverage statistics for EmONC facilities (7, 8). UNFPA is currently in the process of supporting the measurement of the EPMM indicator in several initial countries. In order to model population coverage as realistically as possible, UNFPA is encouraging the organization of travel scenario workshops (TSWs) aimed at estimating the mode of transport and speed of seeking health care, so that travel times can be estimated as realistically as possible.

### 1.1. Knowledge elicitation travel scenario workshops

Over the past decade, the WHO open-source AccessMod software has often been used to model geographic accessibility coverage of various types of health services (11–15). To analyze the accessibility coverage of a country or region, AccessMod applies a least-cost path algorithm to compute the routes with the shortest travel time between any location and the nearest health service, for example an EmONC facility (10, 16). Next to spatial input data on land cover, road networks, elevation, barriers to movement (e.g., rivers, lakes), population distribution and the location of health facilities, AccessMod needs a travel scenario to model accessibility (10, 16). A travel scenario aims to capture the health seeking behavior of the target population's, e.g., the people who need to benefit from the health services, by providing information about their means of transport and speeds according to the type of landcover or road. For example, a travel scenario can reflect that pregnant women in need of EmONC use a combination of walking or being carried on grassland with an average speed of 2.5 km/h to the nearest road, then use motorized vehicles at 40 km/h on secondary roads and 60 km/h on primary roads. This particular AccessMod input travel scenario has a strong impact on the extent of EmONC catchments (i.e., accessibility coverage) and therefore on the calculation of the number of people falling within these catchments (12). This illustrates why it is important for a travel scenario to represent reality as closely as possible. However, to calculate travel time, many accessibility modeling studies use (a combination of) generalized—often unvalidated—travel scenarios that sometimes do not even represent the country of interest (17–28). Consequently, when comparing such model-outcomes to patient reported travel times, serious discrepancies—mainly underestimations on the side of the computed travel times—come to light (17–19).

To develop travel scenarios that are as close as possible to the local context, UNFPA and the University of Geneva (UNIGE) have developed and applied a methodology in -14 countries since 2018- that incorporates local expertise to develop travel scenarios for EmONC accessibility modeling that are tailored to the region of interest (29). To help local experts recall and articulate relevant information, knowledge elicitation TSWs are organized, inviting maternal health-, cartographic-, GIS- and transport experts; local health personnel; various district and health directors; regional or country representatives, and (delegates from) the Ministry of Health (MoH) (29). The activities in the TSWs allow for discussion among the experts, to achieve an inter-validated consensus on the elicited knowledge regarding travel modes and speeds of the target population. A typical TSW lasts between half a day and a day, usually as part of a week-long regional EmONC prioritization workshop (29). Several TSWs are typically run for a given country, each focusing on three to five sub-national regions with representative experts from all areas in the region. Experts from the sub-national regions discuss together as part of a sub-national focus and propose modes and speeds of travel for each road type and off-road landcover type, as well as potential barriers to movement.

The TSW methodology has evolved over time with experience from the 14 countries where it has been applied. Although the original TSWs are considered to have resulted in realistic scenarios and contributed to high quality accessibility models, improvements should be made to optimize knowledge elicitation procedures and the understandability of the concept. Anecdotal evidence has shown, for example, that participants found it difficult to grasp the concept of speed estimation and to read maps, while facilitators found it difficult to communicate and explain the concept of speed in different terrain. Despite existing and increasing improvements in methodology, TSWs have never been evaluated or standardized.

Since accessibility modeling is increasingly used to help improve access to EmONC and to achieve the global recognized EPMM target (7) for 2025, it is key to evaluate the existing expert knowledge elicitation practices for developing realistic travel scenarios. Therefore, to identify the main barriers for TSW participants and to understand how TSW facilitation can be improved, this article explores strategies to optimize and harmonize knowledge elicitation in TSWs by inclusively evaluating the past regional TSWs that have been part of UNFPA's EmONC development approach under leadership of ministries of health and governments. This will not only add realism to the travel scenarios, but will also help participants and facilitators in future TSWs and would serve as a first step toward standardization. In addition, the study aims to contribute to the co-development of a standard operating procedure (SOP) for future knowledge elicitation travel scenario workshops, which is broadly applicable in the field of geographic accessibility modeling.

### 1.2. Program description

Since 2015, UNFPA has been developing an approach to support governments and ministries of health in their leadership role in improving access to EmONC through the development of national networks of maternity units to achieve target 3.1 of the Sustainable Development Goals (SDGs): reducing the global MMR to <70 maternal deaths per 100,000 live births in 2030 (1, 29–31). The approach finds its origins in 2009, when the national standard for all countries to have “at least 5 EmONC facilities per 500,000 population” was introduced (32). Counterintuitively, it appeared that in countries with a high MMR the number of planned EmONC facilities was often much higher than the minimum recommended standard. In practice this meant that limited resources had to be distributed over more locations, causing the average number of functioning EmONC facilities to be 10-to-30% below the actual recommended standard, with even lower estimates for those facilities providing the recommended quality of care (29). The UNFPA EmONC development approach addresses these issues by following a 6-step guideline which ensures strategic planning, implementation, monitoring and empowerment at the local level (29). Step 3 is concerned with modeling of geographic accessibility coverage—including TSWs—and prioritization of EmONC facilities (29, 33). When the prioritized (regional) networks of maternity units are identified—informed by the EmONC geographic accessibility coverage models—a country's MoH has all the required input data to plan for a functional national network of EmONC facilities (29).

Between 2016 and 2021, the evolving UNFPA EmONC development approach was already implemented under leadership of ministries of health and governments in 14 African and Southern Asian countries: Togo; Burundi; Benin; Republic of Guinea; Senegal, Madagascar, Sudan, Republic of Congo (Sangha and Lékoumou); Ivory Coast; Chad; Burkina Faso; Democratic Republic of Congo (Maniema); Timor Leste; and Niger. Based on these implementations a guideline was developed to standardize the program's workflow (29). However, the TSW methodology was not addressed in this standardization process.

## 2. Methods

Accessibility modeling to support EmONC network optimization following the UNFPA approach has been applied in the 14 countries indicated above. Here we discuss the evaluation of the different TSWs that have been applied in this process by systematically studying the experiences of different involved stakeholders.

### 2.1. Transdisciplinary research design

**Figure 1:**
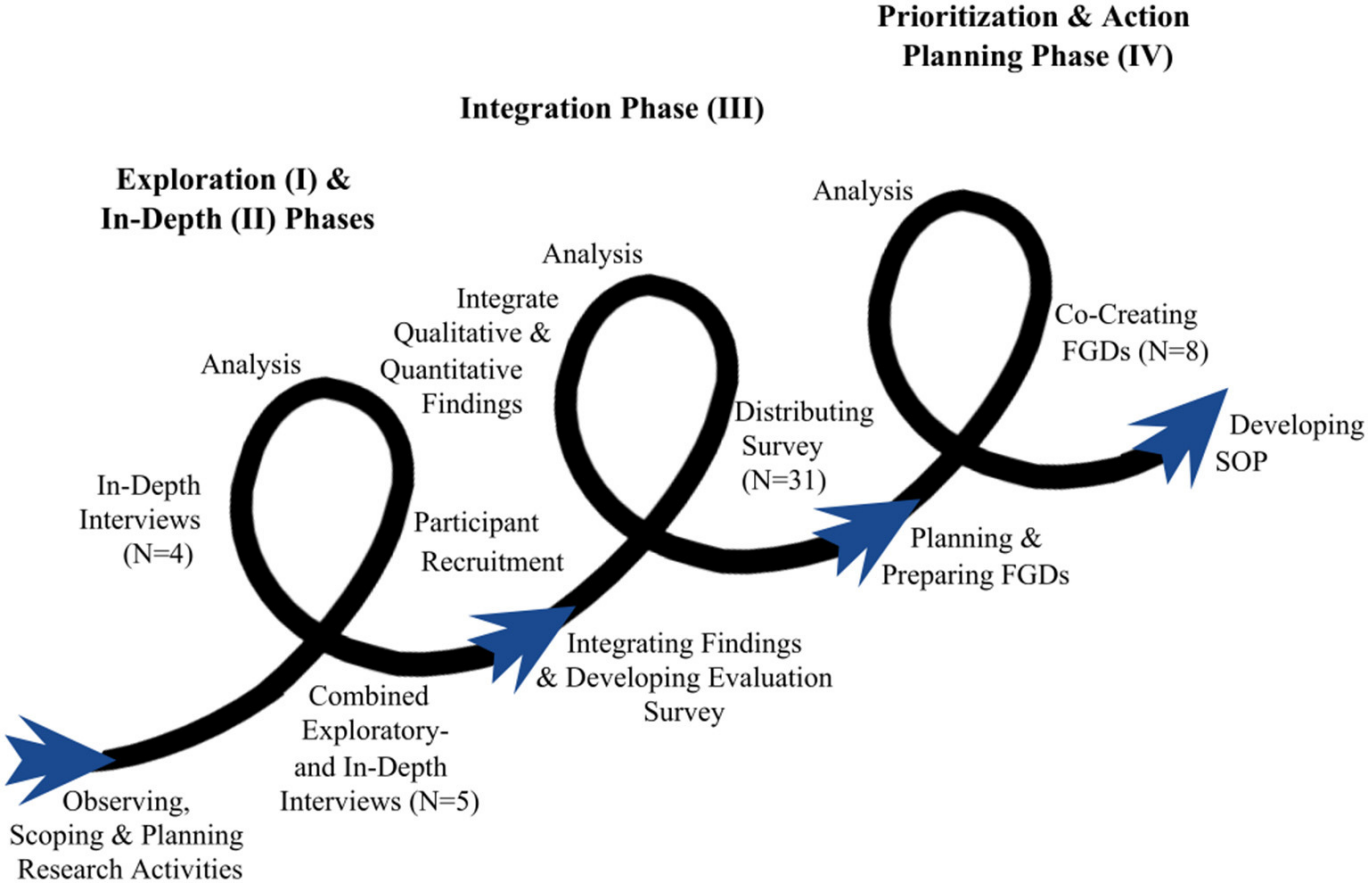
Learning action spirals of the transdisciplinary approach. Each individual curve indicates the separate phases of the approach. The research activities undertaken are shown around the curves. The spirals point to the feedback loops between the different research activities and show how all activities are interconnected and benefit from each other.

In order to evaluate the TSW experiences, barriers and opportunities in a scientifically standardized and robust manner, this mixed-methods evaluation study adopted a transdisciplinary approach using the Interactive Learning and Action methodology (ILA). This was done to facilitate knowledge integration between researchers and local actors to optimize and harmonize TSWs. The ILA methodology—developed and validated by the Athena Institute, VU University Amsterdam—is characterized by five phases: (I) exploration, (II) consultation, (III) integration, (IV) prioritization and action planning, and (V) implementation (34, 35). These successive but overlapping phases help structure the iterative- and learning-action-spiral nature of a transdisciplinary research approach, as the output of each phase serves as input for the next (34–38). The learning-action-spirals with the corresponding phases of this evaluation study—including planning, acting, observing, and reflecting research activities—is shown in Figure 1 and Supplementary Table 1, and are elaborated in the paragraphs below.

Throughout the ILA process, in-depth interviews, focus group discussions (FGDs) and a survey were the key components to assess past TSWs and evaluate lessons learned. The first phase of the study was centered around five combined exploratory and in-depth interviews with invited program level stakeholders who together attended TSWs in all 14 countries. Based on the results of these interviews, additional stakeholders were identified and enrolled into the study through a sequential snowballing process. The next phases included in-depth interviews and surveys with the recruited key stakeholders to evaluate the TSW experiences, barriers, and opportunities. The last learning cycle was focused on FGDs, which were organized with different TSW end-user groups (i.e., organizers, participants, coordinators). Participants of the co-creating FGDs were mixed so that integrated learning was promoted.

#### 2.1.1. Setting and study participants

Data collection and analysis took place between November 2020 and June 2021 and included a workshop-observation, scoping activities, individual interviews, an online survey, and two co-creating FGDs. In general, study participants can be divided into program level stakeholders and country-level stakeholders and were represented by stakeholder groups as follows: Program Level Stakeholders [UNFPA Headquarters, UNFPA Regional Offices, UNIGE], Country Level Stakeholders [Ministry of Health, Regional (Health) Directors, other Country/Regional Representatives, UNFPA Country Offices, GIS & Cartographic Experts, Maternal Health Experts, Transportation/Road Experts, Local Health Personnel, Local Universities].

**Figure 2:**
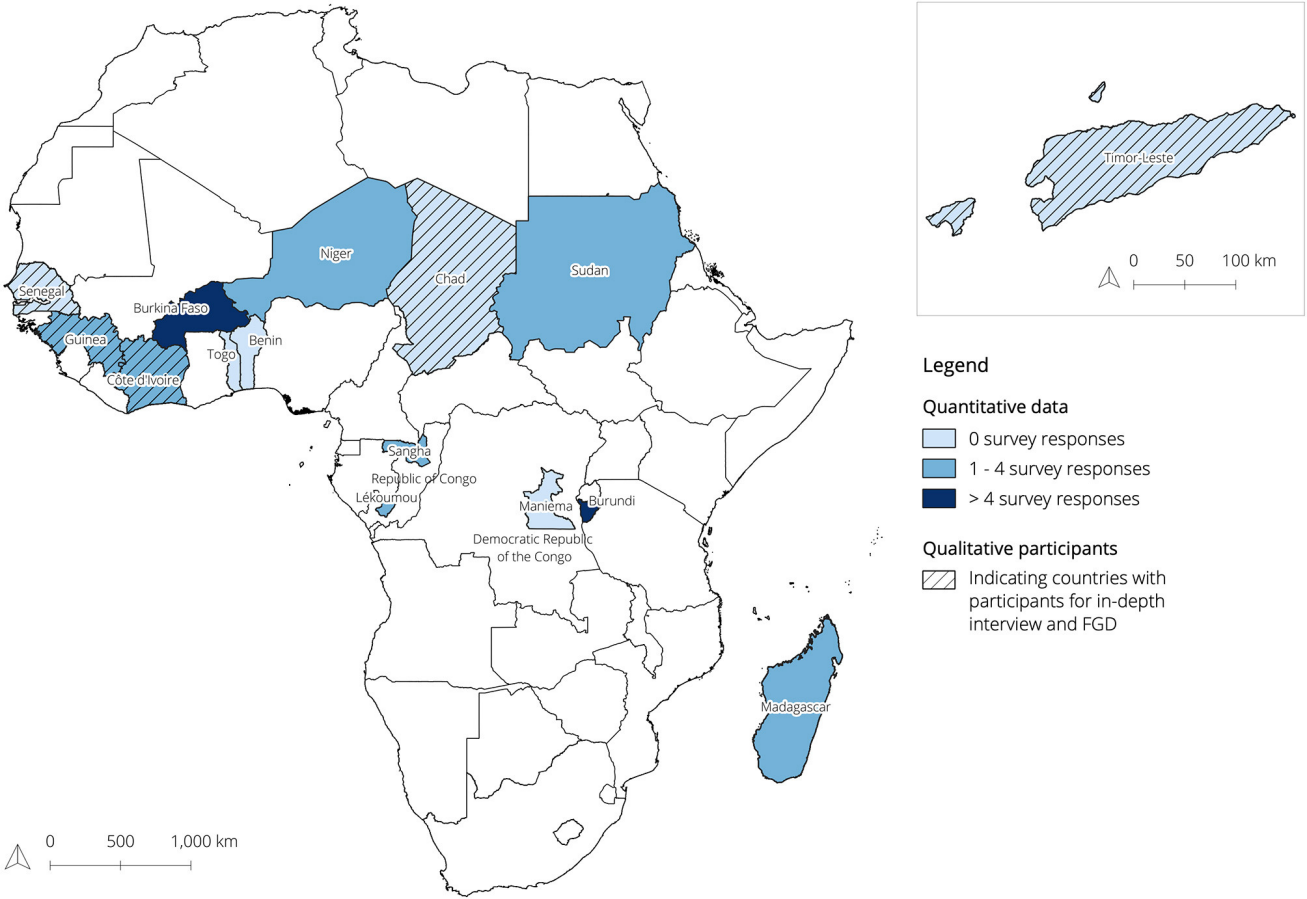
Map showing where the UNFPA EmONC program was implemented by 2021, while indicating where survey (quantitative) and in-depth interview and FGD (qualitative) participants attended the TSWs. The blue color gradient indicates the number of survey responses. Dashed countries indicate countries where the in-depth interview and FGD participants attended TSWs. The combined exploratory- and in-depth interview participants together covered for TSW attendance in all 14 indicated countries.

Program level stakeholders represent individuals who are directly working on the coordination and strategy of the UNFPA EmONC program at the global level. Country or regional level experts represent people who either lead, participate in, implement or execute optimization of the EmONC network at the local level. To ensure a comprehensive assessment of TSWs, the relevant study participants included experts who had participated in one or more TSW workshops as part of the EmONC optimization that was led and initiated by the ministry or government and supported by UNFPA. In total, 40 different study participants with 13 different nationalities and 11 different employment groups were included in the study (Figure 2; Table 1). Of the 40 study participants included in ILA phase I, II, and III, eight were selected by convenience sampling to participate in a FGD (phase IV).

**Table 1:** Characteristics of the study participants (*N* = 40).

		***N***	***N*** **per phase**		
		**Total**	**I/II**	**III**	**IV^a^**
Sex	Male	25	6	19	6
	Female	15	3	12	2
Age	Median	43	46	42	46
	32–44	26	3	23	3
	45–62	14	6	8	5
Employment	UNFPA HQ	1	1	0	1
	UNFPA regional office	1	0	1	1
	UNFPA country office	11	2	9	2
	UNIGE GeoHealth group^b^	4	4	0	4
	National government	8	0	8	0
	Regional government	4	0	4	0
	Public health facility/hospital	4	0	4	0
	Health district	3	1	2	0
	NGO	1	0	1	0
	Local university	1	1	0	0
	Self-employed	2	0	2	0
First nationality	Burkinabe	7	0	7	0
	Burundian	10	0	10	1
	Congolese	2	0	2	0
	Chadian	1	1	0	0
	French	1	1	0	1
	Guinean	1	0	1	0
	Ivorian	5	1	4	0
	Malagasy	3	0	3	0
	Nigerien	3	0	3	0
	Portuguese	1	1	0	1
	Senegalese	2	2	0	2
	Sudanese	1	0	1	0
	Swiss	3	3	0	3

a All FGD participants (phase IV) were also interviewed (*N* = 7), or filled out the survey (*N* = 1).

b The study participants from the UNIGE GeoHealth group (*N* = 4) together covered for TSW attendance in all 14 countries.

#### 2.1.2. Ethics

Before participation, all identified stakeholders were made aware of the nature and aims of this transdisciplinary evaluation study. To protect their autonomy, participants had to individually provide written informed consent to confirm that they were freely and voluntarily participating in a research activity.

### 2.2. Data collection

In the first phase of the ILA, the *exploration phase*, combined exploratory- and in-depth semi-structured interviews of 55–80 min each were conducted with program level actors (*n* = 5) (Supplementary material 1). The main objective of this phase was to fully understand the UNFPA EmONC development approach and its geographic accessibility modeling process, to develop a targeted transdisciplinary evaluation strategy, and to identify and recruit further participants for the next phases of the ILA.

In the second phase of the ILA, the *in-depth phase*, the purpose was to identify and analyze how the TSWs were experienced by the attendees. A total of three written and one oral in-depth interviews lasting 20–40 min were conducted with previous TSW participants (Supplementary material 2). In the interviews, participants also had the opportunity to share barriers, challenges, facilitators and ideas for improvement strategies.

In phase three, the *integration phase*, we analyzed the findings of the nine interviews with five program level stakeholders and four TSW attendees. The main facilitators and barriers of TSWs were extracted and classified into different groups (e.g., difficulty in understanding the map, language barrier, etc.). This information was then used to create an online evaluation survey (in French and English) -using the Qualtrics software (39)- that was distributed by UNFPA country offices to a larger group of previous TSW attendees (Supplementary material 3). The goal of the survey was to gain a quantitative understanding of the most commonly experienced barriers and opportunities for improving TSWs, and it allowed for consultation of 31 other relevant stakeholders, enabling a more comprehensive assessment approach.

In phase four, the *prioritization and action planning* phase, the results of the survey were used to organize two co-creation FGDs to prioritize the different improvement opportunities for future TSWs with eight participants (Supplementary material 4). The FGDs were held with the original five participants at program level and three additional stakeholders, whereof two could be classified as country level stakeholders, both participating in the first FGD (FGD1) (Supplementary Figure 1). Together with all the data previously collected, this brainstorming facilitated the joint development of the initial SOP for future TSWs.

### 2.3. Data analysis

Data collection and analysis were not necessarily sequential activities, but rather alternating or even embedded, following the learning-action spirals (Figure 1; Supplementary Table 1).

#### 2.3.1. Qualitative data

Qualitative data analysis was conducted using the various interviews at the different ILA stages of the study. The results of the different rounds of interviews were in turn used as input for the following research steps of the ILA. The results of the interviews were first translated into English if they were not originally given in English but in French, and then transcribed. The transcribed texts were analyzed using a developed codebook (Supplementary material 5) in the software ATLAS.ti (40). The ATLAS.ti codebook was developed during the different phases of ILA based on commonly or frequently identified practices and themes. All quotations from the nine individual qualitative interviews that were indicated with codes that depicted information about their experiences and perceptions on TSW activities, barriers, facilitators, and opportunities; were transferred to Excel. Additionally, Sankey diagrams were created to visualize which challenges and opportunities were most emphasized. These diagrams and quotation reports served as the most important input data for the integrated evaluation survey.

Both co-creating FGDs were summarized with the help of the audio-recordings. These summaries included the overall storyline during the FGD, the main discussion points, some specific quotes of participants, and the activity outputs. The analysis of the FGDs mainly focused on newly identified challenges and opportunities, the prioritization activities, and the brainstorm regarding SOP formatting. Since both FGDs followed the same design, the tangible activity outputs were subjected to a comparative analysis in ATLAS.ti (40).

#### 2.3.2. Quantitative data

To be able to discuss survey findings (*N* = 31) in the FGDs, first, univariate descriptive analyses were carried out with the survey data in IBM SPSS Statistics (Version 27) (41). Additionally, bivariate analyses were performed to test for possible associations between the independent variables: gender; age; country of TSW attendance; and area of expertise, and the dependent variables: reported roles during the TSW; willingness to individually fill out a travel scenario; willingness to prepare for the TSW; ability to read maps; difficulty assessment of travel speed estimations; perception on allocated time for the TSW; and perception on the facilitator's quality to support the attendees. Because of the limited N, Fisher's Exact Tests were used, considering a 95% confidence interval (42).

## 3. Results

**Figure 3:**
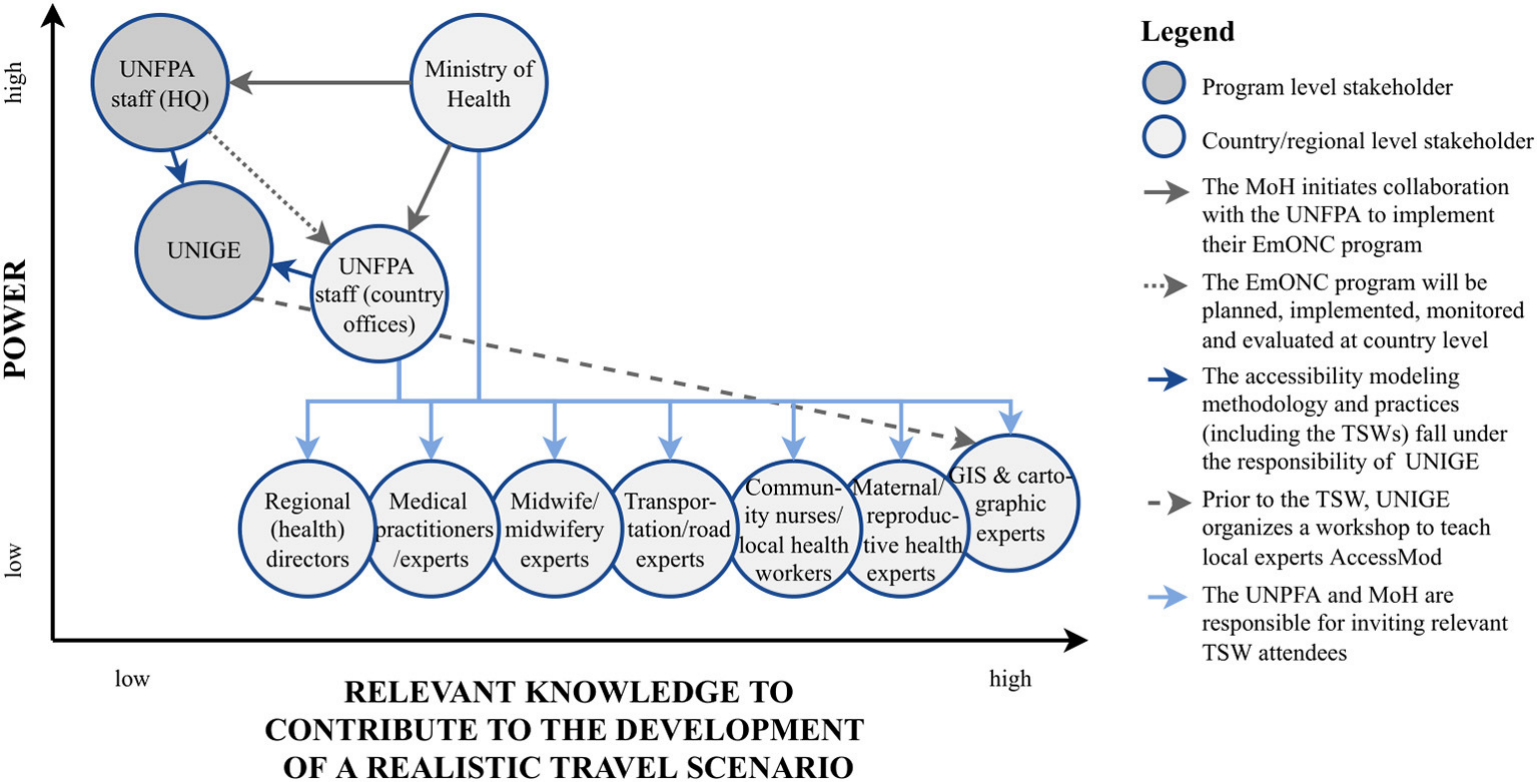
Power-knowledge grid of identified stakeholder groups. The stakeholder division over the power axis is based on the TSW organization within the UNFPA EmONC development approach and further explained/substantiated by the arrows within the figure. The level of relevant knowledge attributed to each stakeholder group is based on the survey results (Supplementary Table 2).

This section provides an overview of the main challenges and opportunities of TSWs as identified and discussed by the wide range of participants who were consulted and involved throughout the ILA process. Based on the combined interviews on the general processes of the UNFPA EmONC development approach and quantitative assessments of relevant knowledge (Supplementary Table 2), a power-knowledge grid was created (Figure 3). The visible tension between the identified stakeholders with the most relevant TSW knowledge and the stakeholders with the power to enforce change should be considered in the rest of this article and forms the basis for the feasibility discussions in the prioritization section.

### 3.1. Challenges

All the barriers and difficulties articulated in the phases of the ILA were summarized in 14 TSW challenges (Table 2). The challenges can broadly be divided into organizational challenges and in-workshop challenges. Organizational challenges can be attributed to the general organization and preparation of the workshops, while in-workshop challenges reflect challenges mostly experienced during the different phases of the workshops.

**Table 2:** Identified challenges.

**Challenge**	**Description**
Facilitator	Having a sub-optimal TSW facilitator
Language	In case attendees do not speak English or French, language is an issue
Methodology	Whether the attendees should/are considering the best/worst/medium case travel scenario
Not up-to-date	The used data/maps/material were not up-to-date
Output control	Not being able to see the effects of the developed travel scenario during the TSW
Participants	The variety and/or number of participants was unsuitable regarding the purpose and/or activities of the TSW
Power	Power, social and/or hierarchical imbalances affecting the TSW activities
Purpose	Difficult to (quickly) understand the purpose and/or utility of the TSW
Reading maps	Participants' inexperience and/or difficulty with reading maps
Remote	Suboptimal TSW activities/outputs because the UNIGE facilitator was only present remotely (due to COVID-19)
Technical	Technical incapability of local computers/devices/connections
Time	Not enough time during the TSW to develop and validate realistic travel scenario's
Travel mode	Difficult to define/agree on the applicable modes of travel
Travel speed	Difficult to imagine/define/agree on travel speeds (km/h) in relation to different modes of transport

#### 3.1.1. Organizational challenges

The organizational challenges included dealing with unfit local equipment [technical], the unavailability of up-to-date data to prepare basic regional maps [not up-to-date], or a possible language barrier [language]. Considerations of whether participants should develop best, worst, medium or multiple case travel scenarios [methodology] were also mentioned. Although time constraints [time] were not the most highlighted barrier in the interviews, 13 (41.9%) survey respondents indicated that the allocated TSW time—usually an afternoon—was not sufficient (Supplementary Figure 2). Furthermore, during a FGD, one UNIGE stakeholder linked the limited time to the inability to show and validate the accessibility models created with the travel scenarios developed [output control]. Due to the COVID-19 pandemic, workshop leaders were unable to travel to conduct some of the TSWs in person [remote], and during some interviews it was mentioned that local facilitators [facilitator] were not optimal. However, as only one survey respondent stated that the TSW facilitator did not fully understand where additional support was needed during the workshop—while 8 (28.6%) indicated to have attended a remote workshop—program level stakeholders did not address this issue further in the FGDs.

#### 3.1.2. In-workshop challenges

**Figure 4:**
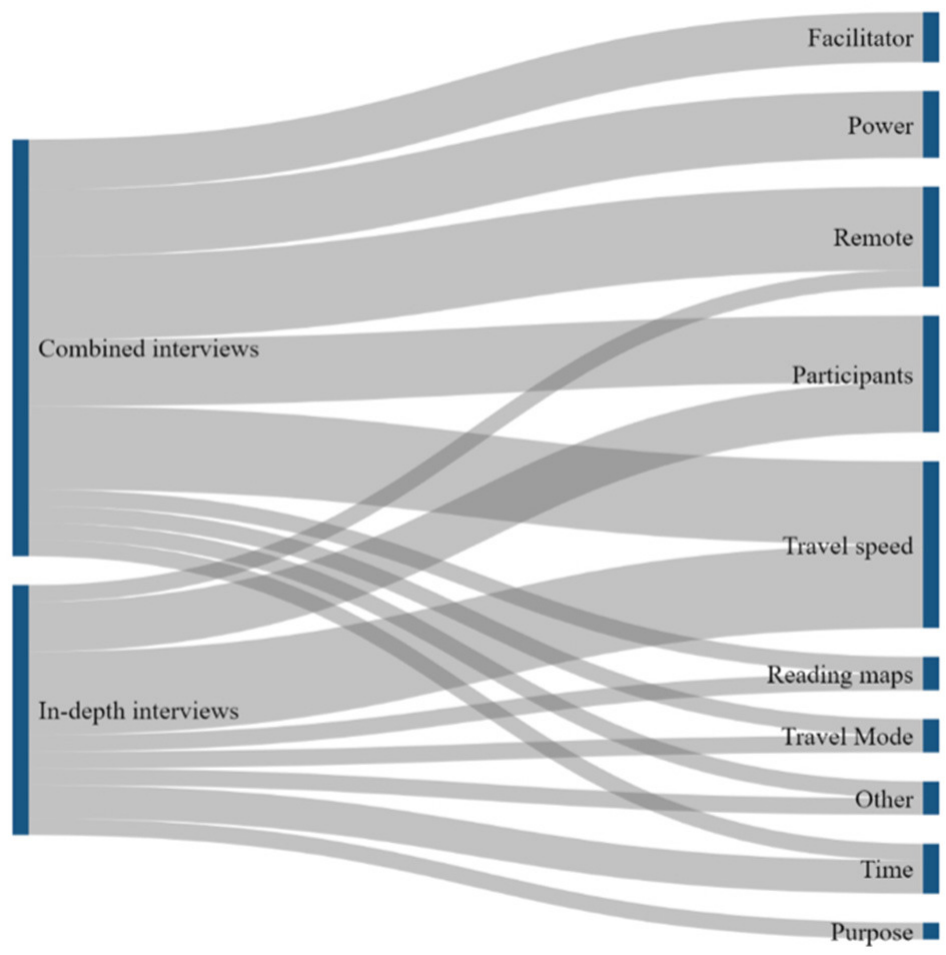
Sankey diagrams showing which challenges were most frequently emphasized by the interview participants.

The remaining challenges identified during the interviews (Figure 4)—power, purpose, map reading, travel mode, and travel speed—were all considered in-workshop challenges. A recurring theme was the ideal number of participants in a workshop and the power dynamics between the participants during the workshop. A favorable number and diverse background of participants during a TSW is a complex challenge, considering that program level actors are not directly responsible for recruitment (Figure 3) as well as that it depends on the availability of invited experts [participants]. Occasionally, countries have specific reasons to invite additional participants. However, a UNIGE accessibility modeling expert specifically emphasized that a higher number of participants did not always contribute to the quality of TSW results.

The interviews also highlighted challenges related to power dynamics between TSW attendees [power]. Yet, the survey results showed that only one survey respondent (3.2%) felt unheard during the TSW. However, 5 (16.7%) indicated that not everyone contributed equally to the development of the travel scenarios, and 22 (71.0%) indicated that a natural leader emerged during the group work (Supplementary Figure 3). No significant relationship was found between gender or age and participants reporting to present the results of the group work during the workshop's plenary discussion.

While the challenge of map reading was mentioned only in passing in the interviews, survey respondents highly supported the statement, “I thought it was difficult to understand the maps of my region/country”, 3 (9.7%) strongly agreed, 6 (19.4%) somewhat agreed and 3 (9.7%) said they were neutral. In addition, 15 (51.7%) respondents said they would have liked to have familiarized themselves in detail with the objectives, terms and materials before the TSW, while another 7 (24.1%) said they would have liked to have prepared briefly [reading maps]. Of the remaining 7 (24.1%) respondents who indicated that the introductory presentation was sufficient to familiarize themselves with the workshop materials, 4 (57.1%) were cartographic experts, resulting in a significant relationship between this type of expertise and the feeling of not needing to prepare for the TSWs (*p* < 0.05).

Finally, the challenges related to the estimation of travel modes and speeds [travel speed; travel mode] were identified throughout all research activities. Figure 4 illustrates the emphasis on the estimation of travel speeds throughout the interviews, explained by, among others, the following statement:

**Quote:**

“*Estimation of travel speeds according to means of locomotion is the most difficult aspect of the workshop.”*

(UNFPA country office, maternal & reproductive health expert II)

In the survey, the difficulty of estimating travel speed was rated on a scale of 0–10 by the participants, with 10 indicating that it is completely impossible and 0 indicating that it is easy and straightforward. The results showed a median score of 5 (Supplementary Figure 4). GIS experts were found to experience significantly more difficulty (scores from 1 to 3) in estimating travel speeds (*p* < 0.05), and the results for cartographic experts also tended in that direction (*p* = 0.055). No significant association was found in relation to any other expertise.

### 3.2. Opportunities

The interviews and survey responses also led to the identification of TSW facilitators and improvement strategies, which were integrated into 18 opportunities (Table 3). These opportunities can be classified into direct, transcending, and travel speed opportunities.

**Table 3:** Identified opportunities.

**Opportunity**	**Description**
Clusters	To develop a travel scenario, subdivide regional stakeholders in smaller groups or group regions with similar characteristics together, allowing for cross-validation
Detailed maps	Having more detailed maps
Facilitator	Having a strong/charismatic TSW facilitator
Field	Collect relevant field data before the TSW
GPS	Use Global Positioning System (GPS) data/trackers to illustrates local movement behaviors
Local training	Realize an actual/more elaborate training on accessibility mapping for local (GIS) experts/students
Participant preparation	Ask the participants to prepare themselves for the TSW (and providing them with the means/materials to do so)
Participants	Make sure to have the right TSW attendees regarding relevant knowledge
Predefined scenario	Use/have/show (afterwards) a predefined travel scenario in the TSW
Referral	Use referral information on traveling between facilities to define travel speeds
Road experts	Having a road (network) expert present during the TSW, with particular knowledge of the current road statuses regarding the area of interest
Technical	Improve local access to technology/capable devices
Time	Having more time for the TSW to develop and validate realistic travel scenarios
Travel time	Ask the workshop participants to define travel times, from which you can then calculate the travel speeds
TSW preparation	Involve relevant workshop participants regarding the context of the particular TSW (such as the local road/GIS/cartographic experts) in the preparation of the TSW
Uncertainty	Take up uncertainty measures/indications in the end model
Validation	Having the room/tools to (cross)validate the developed travel scenario(s)
Visualizing	Use maps/data/models/photos to visualize the road and speed situations

#### 3.2.1. Direct opportunities

Some of the opportunities are a direct response to the identified challenges, such as having a strong facilitator [facilitator], inviting attendees with relevant knowledge [participants], arranging a suitable environment and adequate equipment [technical], and providing more detailed maps of country or region concerned [detailed maps]. The latter was mentioned in an in-depth interview, in which the maternal health expert from UNFPA's country office explained that he had seen participants struggle with the maps and was convinced that more detail—for example, by indicating more well-known landmarks—would have made it easier for the participants to understand what they were seeing. His view was largely shared by survey respondents, with 9 (29.0%) somewhat agreeing and 16 (51.6%) strongly agreeing with the statement “I would have appreciated it if the country/regional maps had been more detailed”. In the FGDs, this simple need came as a surprise to most stakeholders at the program level.

#### 3.2.2. Transcending opportunities

**Figure 5:**
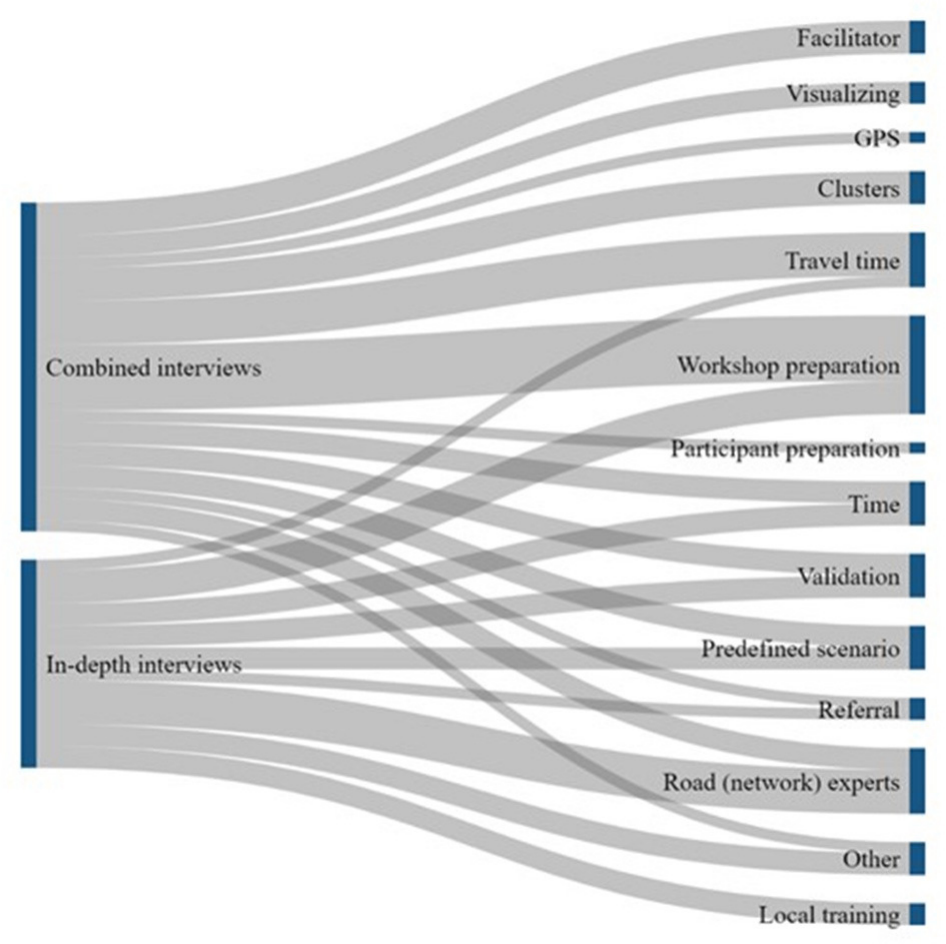
Sankey diagrams showing which opportunities were most frequently emphasized by the interview participants.

The most strongly identified cross-cutting opportunity was the integration of preparation materials to enable TSW participants to prepare themselves before the workshop begins [participant preparation]. This improvement strategy was highlighted in a combined interview with a UNIGE stakeholder (Figure 5) and confirmed by 22 (75.9%) positive survey responses to the statement on whether respondents would have liked to prepare themselves if they had been provided with appropriate resources and materials. If local experts were already somewhat familiar with the TSW objectives, agenda and materials, this could save some explanation time during the introduction to the workshop, giving participants more time to work on and validate the travel scenarios without increasing the overall TSW duration. This reasoning was quickly adopted by stakeholders at the program level. It was also discussed during the FGDs that if all participants prepared, the initial level of understanding would be somewhat neutralized, meaning that the problem of power would be addressed simultaneously. During the FGDs, [TSW preparation] mainly referred to the prior involvement of local GIS, cartographic and/or road (network) experts. These experts know the reality on the ground, which enables them to support the other TSW participants. The survey results also indicated that these experts—compared to the workshop facilitators—more often helped the participants to understand the meaning and relevance of “road types” and “road conditions” (Supplementary Table 3). The participation of road experts in TSWs was also recognized as an opportunity in the interviews: initially road experts were not necessarily invited to the workshops. However, their presence proved beneficial for the development of realistic travel scenarios.

#### 3.2.3. Travel speed opportunities

The remaining opportunities were all formulated in the context of estimating realistic travel speeds. Considered strategies included for example the use of photo or video material to clarify the instructions [visualizing], deploying new- or using existing GPS trackers to illustrate movement behaviors [GPS], or using available data on referral times between health facilities [referral]. While the use of referral times was found to only makes sense in an emergency situation due to the use of motorized vehicles and ambulances in this context, asking participants about travel duration instead of travel speed was found to be promising and feasible [travel time].

Finally, it was also suggested to use a predefined travel scenario [predefined scenario]. For some of the study participants this seemed to be the ideal solution, but others did not agree, which led to a discussion in FGD1. Here, the proponents assume that pregnant women should travel at a similar speed with the same land cover and altitude, regardless of their place of residence or culture. Additionally, they mentioned that a predefined scenario could be useful for estimating road speeds as well, for example based on road categories in neighboring countries. This means that TSW participants can simply agree or disagree with the predefined scenario, which would also save time. However, this is exactly the point that opponents have difficulties with, because it prevents participants from thinking for themselves, which could lead to distortions.

When introducing this discussion point in FGD2, participants agreed that a predefined scenario could be useful as a validation tool for the facilitator to see—and possibly respond to—whether participants are developing a realistic travel scenario. Another validation tool is the possibility to form “clusters” during the TSW [cluster]: At least two clusters develop a travel scenario for the same region(s), whose results can then be compared for cross-validation. 12 (46.2%) of the survey participants indicated that this strategy had already been successfully used during the TSW they participated in.

### 3.3. Prioritization

In order to select the identified opportunities that have the greatest potential for improving TSWs, the different opportunities that emerged from the interviews, survey and FGDs were prioritized according to their degree of facilitation and feasibility.

#### 3.3.1. Level of facilitation

**Figure 6:**
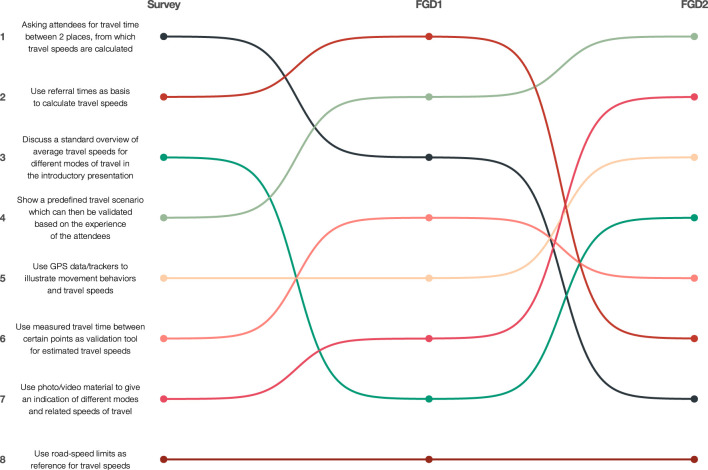
Weighted average-ranking of most promising strategies to optimize travel speed estimations by survey and focus group discussions. Colors indicate separate strategies. Lines indicate the changes in ranking position respective of the survey and focus group discussions.

Both the survey and FGD participants were asked to rank eight formulated improvement strategies related to the difficulty of estimating travel speeds, where number one indicated the most promising strategy to address this issue. It is assumed that the highest ranked strategy is the most beneficial for optimizing TSW results and therefore has a high degree of facilitation. The results of the ranking did not agree very well with each other (Figure 6). This discrepancy can likely be explained by the different perspectives of the various stakeholder groups. In FGD2, where only two program-level stakeholders participated in this activity, it became clear that the focus was on obtaining the most reliable estimate of travel speed and providing TSW participants with the best material to make an informed decision, while TSW participants focused on the strategies that would lead to a practical simplification of this estimate. Despite these discrepancies, both “Asking attendees for travel time between 2 places, from which travel speeds are calculated” and “Use referral times as basis to calculate travel speeds” were ranked in the top-3 of most promising strategies in the survey and FGD1- consisting of a mix of program level and country level stakeholders - rankings and could therefore be a promising way forward for future TSWs. In FGD2 the discrepancy between their average ranking and the survey results was seen as a logical consequence of their different perspectives. This reasoning would explain why survey respondents ranked the use of travel time 1st—while this came 3rd and 7th, respectively, for FGDs—and why FGD2 anticipated a high level of facilitation in the use of photo and video materials (2nd), while this came 7th for survey respondents. Despite the lively discussion about the use of predefined travel scenarios in FGD1, this possible validation tool was ranked 2nd—and even 1st in FGD2—while it was only ranked 4th by survey respondents.

#### 3.3.2. Level of feasibility

**Figure 7:**
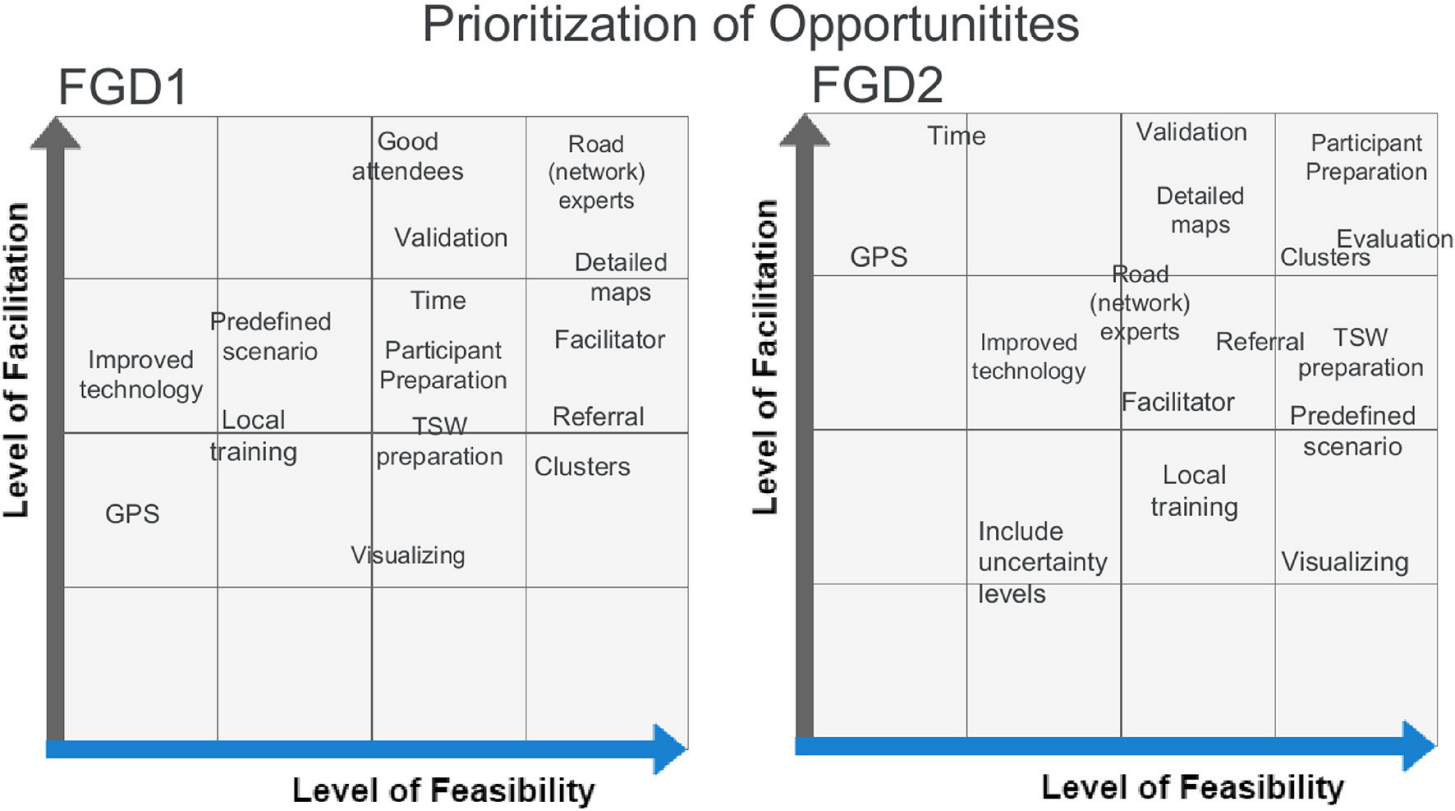
Facilitation-feasibility grid for the prioritization of identified opportunities. The left grid shows the results for focus group 1 while the right grid shows the results of focus group 2. Each identified opportunity is ranked according to the mutually agreed level of facilitation and feasibility.

The feasibility of the identified opportunities to improve TSW was assessed in the FGDs using a prioritization grid. High feasibility was for example attributed to the use of more detailed maps during both FGDs. Other opportunities that were rated similarly in FGD1 and FGD2 were referral, facilitator, TSW preparation, local training, validation and visualization (Figure 7). The latter was assessed as quite feasible but was not expected to significantly improve TSW results. Validation of travel speeds and modes seemed to be quite high in both grids and could be strengthened, for example, by creating clusters, which was assessed as very feasible in both FGDs. The lowest feasibility scores were attributed to increased access to local technology and the use of GPS trackers. However, compared to FGD1, program-level stakeholders in FGD2 felt that GPS trackers would be a great facilitator, as also shown in Figure 6. The inclusion of uncertainty levels around speed estimates was a new possibility identified in FGD2 but was rated lowest on their prioritization grid.

An apparent discrepancy in the feasibility assessment can be seen for predefined travel scenarios. A discussion arose in FGD1 on how to develop and validate a representative predefined travel scenario: Most accessibility modeling experts felt that this would not be a problem due to the available data from the previous implementations of the EmONC program, but not everyone agreed. Interestingly, using road-speed limits as a reference for travel speeds was considered bad practice in both the survey and FGDs. Finally, evaluation was newly mentioned as an improvement strategy at the end of FGD2 and was high on their grid. Evaluation of TSWs in particular did not take place prior to this comprehensive evaluation study, but the new lessons learned from the FGD convinced participants that this should become a regular practice in light of future TSWs. As the TSW evaluation study has already been developed, the feasibility of this opportunity is high.

#### 3.3.3. Standard operating procedure

To co-create a SOP regarding TSWs, the FGD participants brainstormed about the possible format. All present stakeholders agreed that a thematically organized checklist seemed the most simple and effective way forward. Additionally, in FGD2 it was suggested to emphasize the strategies that are anticipated to have the most effect considering optimization of travel scenario outputs, for example by indicating them in **bold**. Following these formatting ideas and the inclusive prioritization of opportunities, an initial SOP was created, which should be seen as an addition to the normal TSW structure (Supplementary Figure 5).

## 4. Discussion

This transdisciplinary evaluation provides rich data on how the different identified stakeholders thought the TSWs could be optimized and harmonized in the context of the UNFPA program to improve access to EmONC. On the one hand, the country level stakeholders—who usually attended 1 TSW—seemed to focus more on direct strategies to help guide them through the TSW. They for example stressed the possibility of allowing them to prepare for the workshops, using more detailed maps during the introduction, being asked about travel times instead of travel speeds, or having more time to develop the travel scenarios. They also would have liked to be provided with more cues on what they should be thinking of in terms of travel speeds.

On the other hand, program level stakeholders—who mostly organized and facilitated TSWs in multiple countries—were at first much more focused on factors of which they had experienced benefits in a particular TSW, compared to others. Although during the FGDs the program level stakeholders showed to be highly receptive of the attendees' TSW evaluation-results—they were for example very open to the idea of preparation, both on the organization and attendees-side—in general they maintained a more program-oriented perception with regard to the opportunities. This means that their emphasis was on providing the best possible information for the attendees to work with, but without introducing possible bias and while taking resource availability (feasibility) into account.

Yet, they also acknowledged that recording health seeking behaviors in terms of travel modes and speeds was a complicated task for the TSW attendees, and that this could perhaps be addressed with the help of predefined scenarios, by using referral times, or by asking for travel times. In a study focused on access to surgical and anesthesia care in the Pacific Region it was found that timely accessibility could best be based on local travel time knowledge as opposed to internet-based maps or by satellite-informed population density data: In 5 out of 14 assessed countries, the within 2-h travel time access-radius around health facilities was determined by elicited expert knowledge instead of geospatial modeling (43). Additionally, in studies in France and Sierra-Leone, patient-reported travel time to health facilities was used to check whether modeled travel times—based on standard travel scenarios—approached reality (18, 19). However, van Duinen et al. (19) also questions the reliability of this reference data in terms of punctuality and reproducibility, for example because time perceptions can be influenced by cultural factors or surrounding events (44, 45). Besides, AccessMod and other similar tools (e.g., *costdistance* tool in ArcGIS) still requires travel speeds as input data to model geographic accessibility coverage of health facilities, meaning that a tool or strategy is required to translate the articulated travel times into travel speeds, while also taking the travel modes and types of land cover into account.

The issue at hand is that considering health seeking behaviors, no regional experts are trained to specifically assess travel time or speed. Local health personal might have the best view on where (pregnant) mothers reside and where they go when they need treatment. Doctors and health officers may also have clear ideas of the time it takes to move between specific facilities, however they lack particular expertise on maps, transportation networks, or geographic models and their determinants. Contrarily, the local GIS and cartographic experts that were already familiar with these terrains, particularly indicated to have difficulty with assessing travel speeds, possibly explained by their inexperience with the reality of the field. This issue explains the broader academic trend toward more cooperative modeling practices, which shows to improve model outputs, while at the same time already raising awareness on the concerned topic among the engaged stakeholders (46–48).

Notwithstanding the fact that the UNFPA development approach to organize inclusive TSWs is in line with this substantiated trend, in terms of travel speed estimations it does not (yet) seem to be ideal. A possibility would be to take up uncertainty measures in the final accessibility models, as was done a posteriori in some studies (e.g., Curtis et al. and Hierink et al.) (11, 12). However, this strategy would complicate the following steps in the UNFPA EmONC development approach and was therefore not favored. While no literature exists on how to best elicit knowledge about travel speeds, there has been a substantial amount of research on how to best record realistic travel speeds without human involvement. To improve estimations and the distribution of vehicle speeds, studies for example make use of approaches with cameras, loop detectors, radio sensors, advanced sensor technologies, spatial-temporal correlations, vehicle trajectory data, and algorithms (49–53). However, most of these approaches require expensive devices, or need extensive and precise input data on the concerned transportation networks in terms of management, conditions, congestions and deployment (54–57), making them challenging considering that the EmONC program is implemented in low resource settings.

In the context of walking trails, travel time is often calculated based on the length of the route, an average walking speed—as traffic jams or congestions are not very common on trails—and while taking into account possible obstacles and elevation data. For example, Naismith calculated that hikers of reasonable fitness take 1 h to walk 4.82 kilometers considering a flat underground (58). This idea that pedestrians subjected to the same conditions are characterized by a similar speed of movement rhymes with the discussion on the possible usefulness of a predefined scenario considering pregnant women and their walking speed, regardless of their country or region of residency. Still, applying popular functions, such as Tobler's (59) or MIDE (60), to calculate an approximate precise travel time is not an option, because they also require trail-length as input, which is not always easily available as it would require residential addresses or zip-codes of pregnant women, illustrating the utility of AccessMod to model catchment areas (61).

Yet, technological use in the global south should be considered: in 2019, 77% of the people in Sub-Saharan Africa used a sim card connection, whereof 44% was used in a smartphone device, with both shares still rapidly growing (62). Especially smartphones have extensive possibilities considering data collection in the context of accessibility modeling, because they usually include a GPS receiver, pedometer and camera. Various studies acknowledged this opportunity and investigated the use of GPS-based smartphone applications to capture travel behaviors, while checking the reliability (63–65). Additionally, a study in South-Africa found predominantly positive attitudes toward the usage of such an application in the context of continuous engagement in HIV care among peripartum women (66). However, feasibility of the GPS opportunity was considered to be very low in both FGDs. When the GPS strategy was discussed in interviews or FGDs, it always referred to the utilization of trackers: either for motorized vehicles—which may be expensive—or by means of an application as it was illustrated in the examples above, giving rise to ethical challenges. It was however never considered in relation to the attendee-preparation strategy, which might represent a new opportunity: when supplying the attendees with the preparation materials for the TSW, it could also include the suggestion to download a personal tracking application—using GPS or pedometer methodology—or a speedometer application, which can measure the speed of moving objects with the camera. Although these applications might not always be perfectly accurate, by using them in the days before the TSW—on voluntary basis—it may allow the attendees to become a little more familiar with the concept of travel speed. However, the implementation of this might lead to power imbalances, as wealthier or more powerful people are more likely to have smartphones and better access to electricity-, internet-, and phone networks.

### 4.1. Strengths and limitations

This is the first study to our knowledge specifically focusing on optimization of expert knowledge elicitation strategies regarding travel modes and speeds, by evaluating TSWs. The results shine a light on potential improvements for the TSWs, so that access to EmONC facilities and other health services can be modeled more realistically, supporting ministries of health and local governments in the decision-making processes for EmONC network optimization. The most obvious strength of this evaluation study is represented by the application of an inclusive approach informed by the ILA methodology. By means of consulting so many different stakeholders with experiences based on TSWs in 14 different countries, a wide range of knowledge and perceptions was integrated, resulting in the identification, examination, and prioritization of a comprehensive overview of TSW improvement strategies. Additionally, knowledge co-creating was facilitated by means of the two co-creating FGDs.

However, interactive learning and reflection mostly occurred among the program level stakeholders, because they accounted for six out of the eight FGD participants. During the FGDs the participants were made aware of the experiences and ideas of the country level stakeholders by means of discussing the survey results. Ideally, a third FGD would have been organized in addition to the other two. However, due to time constraints this was not feasible under the current timeframe of the study. Another limitation is that some country level experts were only weakly represented among the study participants, such as road experts, local health personnel and delegates of the MoH. Additionally, doctors were not represented at all. Supplementary research is needed to learn about their perspectives on how TSWs can be optimized.

The number of survey responses also represents a limitation: although the response count was higher than anticipated, the *N* was still insufficient to identify possible cultural influences—differences between countries—as well as that very limited associations could be identified with regard to other demographic information and the dependent variables. Finally, regardless of the memory refreshment Supplementary material that was added to all in-depth interviews and within the survey file, recall bias might have played a role for some study participants, considering that in a few countries the TSWs were already organized in 2017 or 2018.

Despite of these limitations, this evaluation study highlighted and prioritized opportunities to enable the development of more realistic travel scenario outputs of future TSWs. With regard to the results, it is recommended to implement the inclusively developed initial SOP in coming travel scenario workshops to measure the EPMM indicator and EmONC population coverage, while closely monitoring and evaluating its impact, with the aim to work toward the development of a final and validated SOP. In this way, both harmonization and optimization of TSWs is addressed, and should be maintained by means of continuous evaluation. Furthermore, it is recommended to dedicate future research to the exploration of smartphone applications as a possible tool to familiarize local expert with travel speeds, considering its possible level of facilitation in the context of geographic accessibility modeling.

## 5. Conclusion

This study showed that the different identified stakeholders had contradicting, complementing and overlapping ideas about strategies to optimize and harmonize TSWs. While country level stakeholders predominantly thought about TSW optimization with the vision of increased local and personal benefits, program level stakeholders also considered the overall goals of the EmONC development approach. The estimation of realistic travel speeds while taking into account the identified modes of transport was found to be very complex for the TSW attendees, causing it to be the key challenge, which remained without an unambiguous solution. Yet, inclusive prioritization of identified opportunities resulted in a consensus that most of the identified challenges—including the travel time challenge—can largely be addressed by means of more extensive preparation, both on the side of the organization (program level) and the side of the attendees (country level). Additionally, after each TSW, evaluation and validation should be stressed. An initial SOP has been co-created stating all relevant strategies that are anticipated to optimize the development of realistic travel scenarios based on expert knowledge elicitation. This SOP is not only relevant in the context of the UNFPA EmONC development approach, but also for monitoring the newly launched EPMM indicator and even in the broader field of geographic accessibility modeling.

## Data availability statement

The data underlying the results will be made available by the authors upon request.

## Ethics statement

Ethical review and approval was not required for the study on human participants in accordance with the local legislation and institutional requirements. The patients/participants provided their written informed consent to participate in this study.

## Author contributions

LM conducted the research, including the design of the study, data collection, and data analysis supported by NR and FH. NR and FH supervised LM throughout the research process. J-PM and MB provided critical feedback and access to participants throughout different stages of the research. LM wrote the initial draft and FH, MB, J-PM, and NR provided helpful comments and revised the manuscript. All authors have read and approved the submitted version.
