# Supplementary material for: Travel scenario workshops for geographical accessibility modeling of health services: A transdisciplinary evaluation study
Source: Front Public Health. 2023 Jan 18;10:1051522. doi: 10.3389/fpubh.2022.1051522 (PMC9889992; doi:10.3389/fpubh.2022.1051522)
Supplement: Supplementary file 1 [file Data_Sheet_1.zip › Supplementary Figures and Tables.PDF]

Supplementary Figures and Tables

Tables

Supplementary Table 1 presents an overview of the research activities per applied ILA phase (I-II-III-IV). Implementation (ILA phase V) was outside the scope of this research.

ILA Phase				Research Activities	Date
I	II	III	IV		
				• Observing the remote TSW in Timor Leste	Nov 2020
				• Scoping meetings with UNIGE staff to affirm the research design and objectives, and establish contacts within UNFPA HQ	Jan-Mar 2021
				• Reviewing the technical country reports of Sudan and Togo<br>• Preparing the combined interviews	Mar 2021
				• **Combined exploratory- and in-depth interviews (N=5)** with the UNFPA EmONC program director and 4 UNIGE AccessMod experts who together attended TSWs in all 14 countries where the EmONC program was implemented<br>• Recruitment of participants for in-depth interviews and FGDs, and finalizing the in-depth interview guide	Mar-Apr 2021
				• **In-depth interviews (N=4)** with 2 maternal- and reproductive health experts, 1 GIS expert and 1 health economist who also had cartographic expertise	Apr 2021
				• Analyzing all individual interviews<br>• Integrating the in-depth findings to develop a TSW evaluation survey for past workshop attendees	Apr-May 2021
				• Distributing the survey to past workshop attendees via 13[a] UNFPA country offices<br>• Recruiting more FGD participants	May 2021
				• By means of the **TSW evaluation survey (N=31)** consulting cartographic experts, GIS experts, maternal- and reproductive health experts, community health workers, nurses, midwifes, regional (health) directors, regional representatives, a road (network) expert, local UNFPA staff, an epidemiologist, a policy development evaluator, and delegates of the MoH	May-Jun 2021

ILA Phase				Research Activities	Date
I	II	III	IV		
				• Analyzing survey responses<br>• Integrating the in-depth- and survey findings to prepare the co-creating FGDs<br>• Organizing **2 co-creating FGDs (N=8)** with UNIGE- and a diversity of UNFPA stakeholders<br>• Inclusively prioritizing possible TSW improvement strategies and discussing ideas about the formatting of a SOP<br>• Analyzing and comparing the perspectives of the FGD participants<br>• Developing a TSW SOP	Jun 2021

[a] No survey-distribution request was sent to the UNFPA Country Office in Timor Leste, because the survey was not available in Portuguese.

Supplementary Table 2. Weighted Average-Ranking of Stakeholders' Level of Relevant Knowledge to Contribute to the Development of Realistic Travel Scenarios as Recorded in the Evaluation Survey (N=31)

Assessment of other Stakeholders	Self-Assessment[a,b]
1. GIS/cartographic experts	1. GIS/cartographic experts
2. Maternal/reproductive health experts	2. Community nurses/local health workers
3. Community nurses/local health workers	3. Maternal/reproductive health experts
4. Transportation/road experts	4. UNFPA staff
5. Midwife/midwifery experts	5. Transportation/road experts
6. (Delegates of the) MoH	6. Midwife/midwifery experts[c]
7. Medical practitioners/experts[c]	6. (Delegates of the) MoH[c]
7. Regional (health) directors[c]	7. Regional (health) directors
9. UNFPA staff	

[a] Only displaying the options as they were given in the question regarding the relevant knowledge assessment of others.

[b] No medical practitioners filled out the survey.

[c] Identical weighted averages resulted in a shared rank.

Supplementary Table 3. Indication of Who Helped the Survey Respondents to Understand the Meaning and Relevance of the Main TSW Concepts (N=31)

	**Stakeholders who helped with understanding (%)**			
**TSW concepts**	TSW facilitator(s)	GIS/road/ cartographic experts	Other TSW attendees	Not applicable
Types of roads	29.0%	48.4%	0%	22.6%
Road conditions	25.8%	51.6%	0%	22.6%
Land cover	32.3%	35.5%	0%	32.3%
Modes of transport	41.9%	29.0%	0%	29.0%
Movement barriers	45.2%	35.5%	0%	19.4%
Travel time	54.8%	32.3%	0%	12.9%
Travel speed	61.3%	16.1%	3.2%	19.4%
Referral time	67.7%	22.6%	0%	9.7%
Catchment area(s)	51.6%	29.0%	3.2%	16.1%
Accessibility coverage	51.6%	29.0%	3.2%	16.1%

Figures

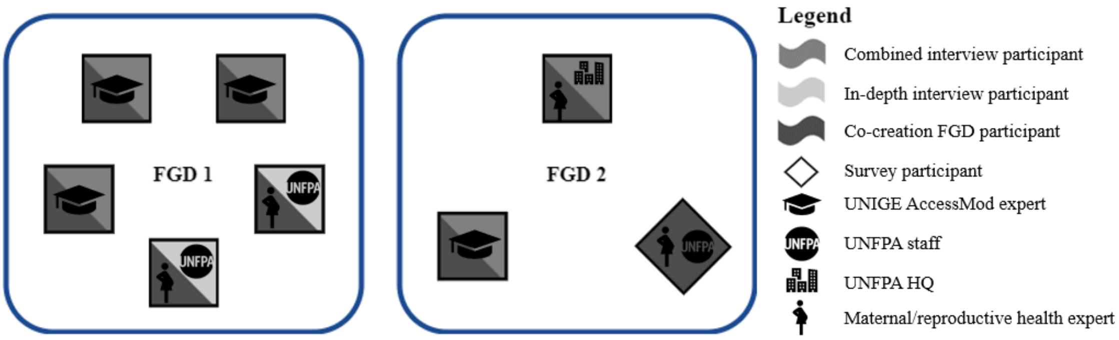

Supplementary Figure 1. Overview of Participants per Co-Creating FGD

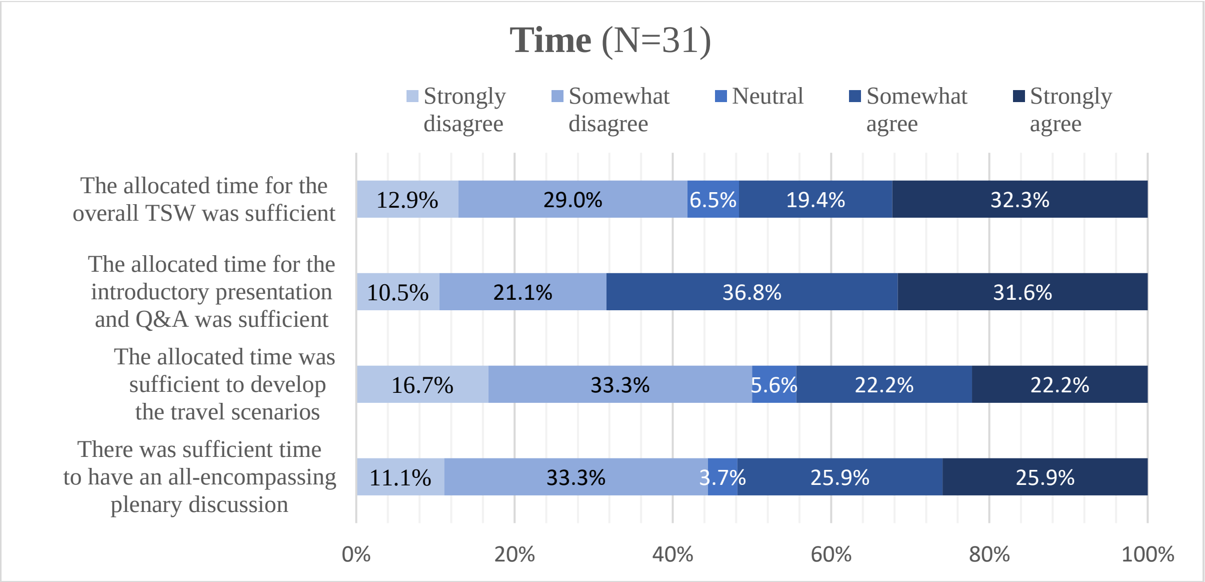

Supplementary Figure 2. Stacked Bar Graph Showing the Survey Responses in Relation to the Identified Time-Challenge

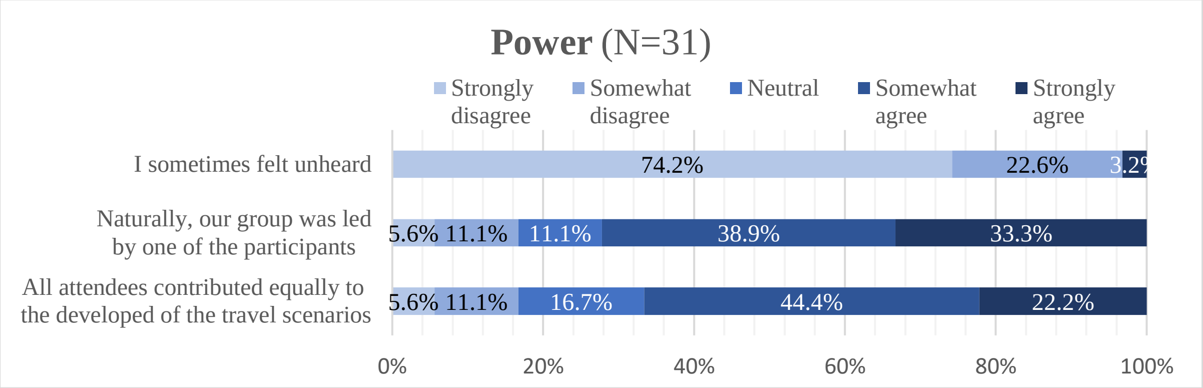

Supplementary Figure 3. Stacked Bar Graph Showing the Survey Responses in Relation to the Identified Power-Challenge

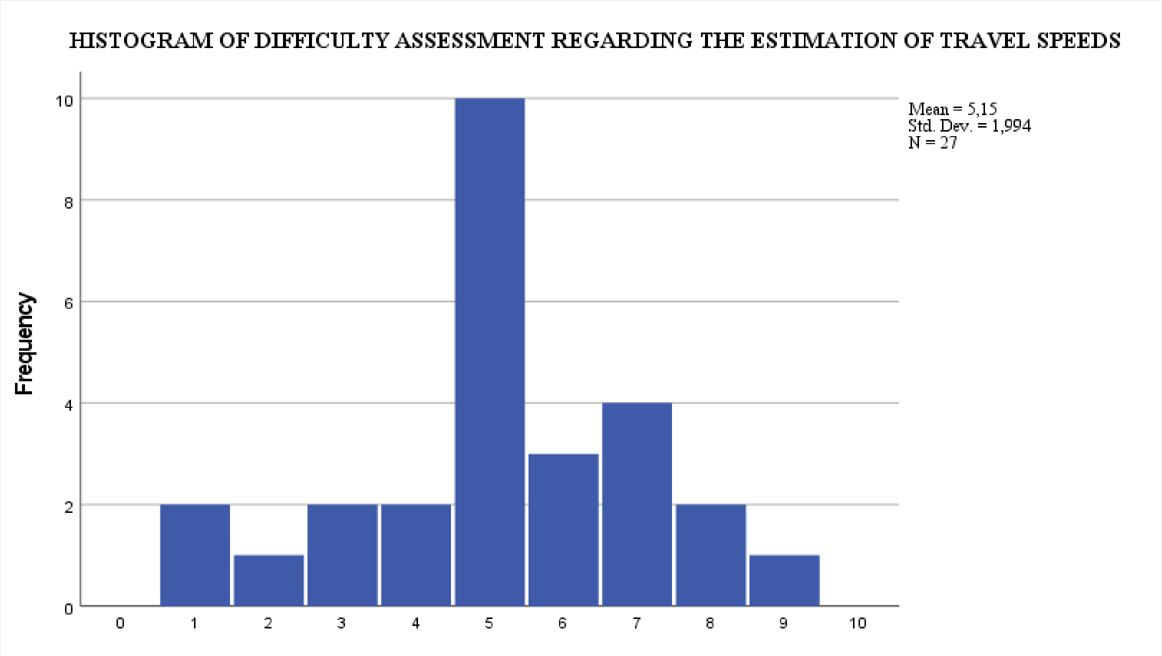

Supplementary Figure 4. Results of the Evaluation Survey's Difficulty Assessment Regarding the Estimation of Travel Speeds

STANDARD OPERATING PROCEDURE

for Knowledge Elicitation Travel Scenario Workshops

in the context of Geographic Accessibility Modeling

Prior to the Travel Scenario Workshop

Overall Organization

- ✓ **Communicate a clear overview of the desired national and local experts to the MoH and UNFPA country office, and stress the importance of their attendance (considering variety and number)**
- ✓ Develop detailed basic maps of the concerned country/region
- ✓ Develop a predefined travel scenario tailored to the applicable context

Inclusive Preparation

- ✓ **Involve GIS, cartographic, and road experts (and, if applicable, local facilitators) in the preparation of the TSW**
- ✓ Develop the attendee-preparation materials together with the above mentioned experts
- ✓ **Require the invited attendees to prepare themselves for the TSW with the supplied materials**

During the Trave Scenario Workshop

Introduction

- ✓ Elaborate and/or illustrate the concept of travel speed more, for example with the help of visualizations, travel times, referral times, or GPS data

Group work

- ✓ **Create clusters based on similar regional characteristics or by dividing experts from 1 region in 2 groups**
- ✓ Make sure the facilitator has access to a predefined travel scenario

Plenary Discussion

- ✓ **Allow room for cross-validation considering the developed travel scenarios between the created clusters**

After the Travel Scenario Workshop

Immediately

- ✓ **Distribute the TSW evaluation survey among the attendees**

As Soon As Possible

- ✓ Verify/discuss the developed accessibility models with the workshop attendees (for example the day(s) after the TSW while the attendees are involved with other EmONC program activities)
- ✓ Analyse the survey results and preferably discuss these inclusively

Supplementary Figure 5. Initial SOP
