# Supplementary material for: Travel scenario workshops for geographical accessibility modeling of health services: A transdisciplinary evaluation study
Source: Front Public Health. 2023 Jan 18;10:1051522. doi: 10.3389/fpubh.2022.1051522 (PMC9889992; doi:10.3389/fpubh.2022.1051522)
Supplement: Supplementary file 1 [file Data_Sheet_1.zip › Supplementary Information 1.PDF]

1. Interview Guide for Combined Exploratory- and In-Depth Interviews

First of all, thank you again for being present during the presentation of my research proposal on the 17th of March, and also thank you for making the time to participate in this interview! As you probably know by now, my name is Lotte Molenaar, and I am evaluating the stakeholder-driven component of the geographical accessibility modeling process and assessments, so as to improve the UNFPA strategy towards increased access to emergency obstetrics and newborn care. Today, I would first like to talk with you about your perception on the overall process of geographical accessibility modeling and assessments. Subsequently, I would like to discuss your experiences with – and ideas about – the travel scenario workshops where you were part of specifically.

As a reminder, this interview will be anonymous and anything that could identify you will be omitted from the transcript in order to protect your privacy. Furthermore, if you no longer wish to participate you may opt out at any time, also when the interview is already finished. Before starting I would like to ask you if I can audio-record the interview? In this way I can transcribe analyze and listen to the interview at a later time as well. As soon as the interview is transcribed I will erase the recording.

< If the informed consent form has not been signed yet, this should be done now.
If not, the interview will not be conducted. >

Opening

1. Ask the participant to tell something about him/herself
    a. Age, career, where are you from?
    b. Since when do you work for GeoHealth/UNFPA?
        i. What does your profession entail exactly?

Geographical Accessibility Modelling

2. How is your profession related to the topic of this research?
    a. From your perspective, what does the complete process of geographical accessibility modelling and assessments regarding maternal health services look like?
        i. How do you experience this process?

ii. What do you think of this process?

3. With which country case studies were you involved?

    a. Were/Are there clear commonalities and/or differences between the different country case studies?

        i. Can you please give some examples? (perhaps considering national policies and politics, bureaucracy, culture, time to completion, and/or other contextual factors)
        ii. Considering this comparison, are there any other things that specifically stood out according to you?

Travel Scenario Workshops (TSW)

4. What actions did you perform prior to the travel scenario workshops?

    a. Considering preparation, liaison with stakeholders to explain the process, etc.

5. In general, how did you experience the travel scenario workshops you were part of?

    a. What is the first thing you memorize thinking back of these workshops?
    b. What other thoughts did you have?

6. What was discussed during the workshops?

    a. Both by the facilitators as among the participants

        i. Please elaborate on any differences between workshops

7. How did you contribute/What kind of actions did you perform during the workshops?

    a. How did you experience this?

8. Did the workshops generally provide for the intended results?

    a. Why or why not?

9. What actions did you perform after the workshop?

    a. Both considering directly after and the process after

TSW Facilitators

10. What do you like about the workshops?

a. According to you, what practices/materials/information work well?

   i. Why?

b. Do you think that the workshop participants experience this in the same way?

   i. Why or why not?

TSW Difficulties/Barriers

11. What do you remember as the most difficult or unclear aspect of the workshop?
    a. Why was this difficult?
    b. According to you, did the participants experience this in the same way?
       i. Why or why not?
12. According to you, were there other aspects that were perceived as difficult by you and/or among the other participants?
    a. Which ones?
    b. Why?

TSW Opportunities/Solutions

13. How would you go about/improve the mentioned barriers/difficulties?
    a. What specific suggestions do you have?
       i. Considering structure/practices/ materials/information?
       ii. What would have helped you?
       iii. What would have helped the participants?
    b. What solution(s)/idea(s) would possibly contribute to a more standardized or harmonized approach and/or outcome(s)?
       i. Why?

Closing

14. After discussing all the previous, how would you (again) reflect on the travel scenario workshops?
15. Is there anything else you would like to ask or add that we have not talked about yet?

Thank you for participating in this interview! I really appreciate you taking time out of your day to talk to me and share your experiences and ideas. If you think of any additions you would

like to tell me, or in case you have contact details of any of the participants of the travel scenario workshops, please contact me (<mailadres>).

Lastly, if you do not mind, I would like to send a short overview-analysis of the interview to you, so you have the ability to read over it yourself and determine whether I have interpreted your ideas correctly. Again, thank you very much and have a nice rest of your day!
