# Supplementary material for: Travel scenario workshops for geographical accessibility modeling of health services: A transdisciplinary evaluation study
Source: Front Public Health. 2023 Jan 18;10:1051522. doi: 10.3389/fpubh.2022.1051522 (PMC9889992; doi:10.3389/fpubh.2022.1051522)
Supplement: Supplementary file 1 [file Data_Sheet_1.zip › Supplementary Information 2.PDF]

1. Interview Guide In-Depth Interviews

First of all, thank you for participating in this written interview! My name is Lotte Molenaar, and I am a Global Health master student at the VU University Amsterdam. In collaboration with the University of Geneva and the UNFPA I am evaluating the stakeholder-driven component of the geographical accessibility modeling process and assessments, to improve theUNFPA strategy towards increased access to emergency obstetrics and newborn care.

With regard to this evaluation study, I believe that your experience with – and perceptions on – the travel scenario workshop(s) provide for valuable insights for this research project. Therefore, I would like to ask you to answer the questions below, considering the travel scenario workshop(s) you attended in <country/region> on <date>. You can answer as extensive or brief as you see fit, but overall it is anticipated that completing this interview takes maximum 30 minutes. In case active memories of the workshop have slightly slipped your mind, please have a look at the attached memory refreshment file.

As a reminder, this interview will be anonymous and anything that could identify you will be omitted from the file before analysis, and the email will be deleted. This measures will protect your privacy. Furthermore, if you no longer wish to participate you may opt out at any time, also when you already finalized and/or sent the interview. Before you start answering the questions, I would like to ask you to check whether you have signed the consent form with your name, as without informed consent from your side, I cannot use the information you provide.

Thank you again and in case of questions you can always reach out to me via <mailadres>

Opening Questions

1. Could you please tell me some things about yourself? (for example your age, where you are from, your occupation etc.)

2. According to you, why and by who were you asked to participate in the travel scenario workshop?

3. Could you shortly mention the course of activities during the workshop you attended?

Experience/Perception

4. What is the first or most striking thing you memorize when you think back to the travel scenario workshop?

5. What information, materials and/or tools were used to help you understand and identify travel speeds considering different roads and modes of transport?

6. What was discussed during the workshop (both by the facilitator as among the participants)?

7. How would you describe your contribution to the workshop?

8. What did you learn from the workshop?

9. In what way(s) have you been informed about the results of the workshop?

10. How would you describe your overall experience of the workshop?

Positive aspects of the workshop

11. What did you like about the workshop? (for example, think about practices, tools and/or activities that worked well or helped you in some way)

12. According to you, would the other workshop participants agree on these positive aspects? (please explain why or why not)

Difficult aspects of the workshop

13. What do you remember as a the most difficult or unclear aspect of the workshop? (please explain why)

14. Were there other aspects that were perceived as difficult by you and/or among the other participants? (please explain which one(s) and why)

Solutions/Improvements

15. How would you change the workshop to overcome the mentioned difficulties? (for example considering the activities or set-up of the workshop, but don't be afraid to think outside of the box either!)

16. Suppose you had every possible means, what solution(s)/improvement(s) would you propose?

17. What is your motivation behind the answers to question 15 and 16?

Closing Questions

18. After answering the previous questions, how would you (again) reflect on your experience of the travel scenario workshop? (in case you would describe your experience different then you did in your answer to question 10, please explain why it has changed)

19. Related to the topic of this research, what would you perhaps like to say to the UNFPA, GeoHealth group and/or Ministry of Health?

20. Is there anything else you would like to add to this interview?

Thank you for participating in this interview! Myself, the UNFPA and the University of Geneva, really appreciate you taking the time out of your day to talk to me and share your experiences and ideas. If you think of any additions you would like to tell, feel free to contact me!

Memory Refreshment of Knowledge Elicitation Travel Scenario Workshops

To refresh your memory of the travel scenario workshop(s), below some clues are presented. However, the exact content and course of the workshop(s) you attended may have been

different. The information below is only meant to refresh your memory a bit, in case it has been a while since the workshop took place. To answer the interview questions, please trust on your own memories of the event. If necessary, explain a bit about the specific activities during your workshop in your interview answers, to clarify the context.

Workshop details

Date: ../../..

Time: ..:..

Location: <address/region>, <country>

Travel scenario for: <region/country>

Goals of the workshop

By means of consensus among the participants, the workshop(s) were intended to:

1) Define the modes of transports, both on and off roads, used by the target population.[i]
2) Determine the average travel speeds on roads as well as off roads, considering the target population.[1]
3) Discuss the possible barriers for movement, and the role of seasonality.

Workshop activities

Although the exact course of activities might have deviated per workshop, roughly seen it included the following activities:

1) Workshop opening by a facilitator.
2) Presentation and explanation of AccessMod and the functionalities of the tool, to demonstrate the relevance of the workshop. To illustrate the story some slides were used, which may have looked like this:

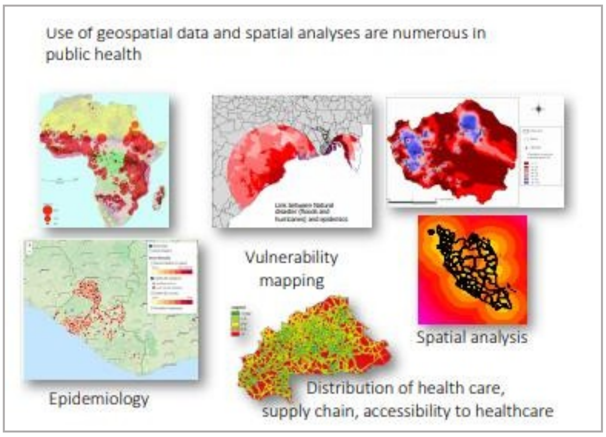

[i] Target population = pregnant women and (young) mothers.

3) Working in groups to define the modes of transport and travel speeds. The outcomesof the group work were (most likely) recorded in a table which may have looked likethis:

Roads	Conditions	Mode of transport (examples are listed below). Please indicate **in bold** the most commonly used mode of transport.	At what frequency does the population use this mode of transport? *	Estimation of average speeds (in km/h), taking into account regional specificities (such as road conditions) **in the dry season**	Estimation of average speeds (in km/h), taking into account regional specificities (such as road conditions) **in the wet season**
Primary roads	asphalted	car			
		motorcycle			
Secondary roads	partially asphalted	walking			
		car			
		motorcycle			
		bicycle			
Tertiary roads	not asphalted	walking			
		animal driven cart			
		motorcycle			
	tracks	walking			
Off-road travel	open environment	animal driven cart			
		walking			
	forest environment	walking			
	agricultural environment	walking			
	rivers	pirogue			
	…	…			

** choose in between: "very often"; "often"; "sometimes";"rarely".*

4) Discussing the outcomes of the group work with all participants.
5) Closing statements by the facilitator
