# Supplementary material for: Travel scenario workshops for geographical accessibility modeling of health services: A transdisciplinary evaluation study
Source: Front Public Health. 2023 Jan 18;10:1051522. doi: 10.3389/fpubh.2022.1051522 (PMC9889992; doi:10.3389/fpubh.2022.1051522)
Supplement: Supplementary file 1 [file Data_Sheet_1.zip › Supplementary Information 3.PDF]

Co-Creating FGD Script

Introduction:

Based on information from the combined and in-depth interviews, and from the survey responses, 2 focus group discussions were organized to discuss the findings, brainstorm about the best approach(es) for future workshops, and categorize and prioritize these ideas. In this way the set-up of the state-of-the-art guideline will be co-created.

Objectives:

To identify the perspectives of the focus group participants on the challenges and opportunities of the travel scenario workshops.

- What are the perspectives of participants on the identified challenges and opportunities regarding the travel scenario workshops?
  - What (other) challenges do participants identify?
  - What (other) opportunities do participants propose?
- Which proposed opportunities are considered to be most promising by the participants, based on feasibility and effectiveness?
- How do they envision a guideline/outline for future travel scenario workshops?

Methods:

Brainstorming and discussion of ideas and perspectives, whereafter these will be categorized and prioritized.

Participants:

- 3 to 5 participants representing various study stakeholders and expertise's (i.e. UNIGE staff, UNFPA staff, maternal & reproductive health experts)
- Recruitment: Through previous interview and survey responses.

Duration:

2 hours

Setting:

Zoom meeting

- Monday the 14th of June, 2021, at 3PM CET
- Tuesday the 15th of June, 2021, at 4PM CET

Facilitator:

Lotte Molenaar

Necessities:

- Zoom
- Jaboard
- Mentimeter
- PowerPoint
- Audio recording via Zoom & back-up device
- FGD Script

FGD Script

Timing	Topics per section	Description of Activities	Objective(s)
Total:<br>15 min<br><br>Start time:<br>15.00<br>End time:<br>15.15	**General introduction**<br><br>- Welcome<br>- Overview<br>- Rules/Respect/ Ethics<br>- Personal introduction(s) + icebreaker	<u>Welcome</u><br>Welcome everyone, and thank you for participating in our FGD. I really appreciate you taking the time out of your day to be here. For the ones who don't know me, I am Lotte and I am doing a research internship at UNIGE with Nicolas Ray, and the coming two hours I would like to learn more about your perspectives on challenges and opportunities regarding the Travel Scenario Workshops.<br><br>>> *Open PowerPoint presentation & share screen* <<<br>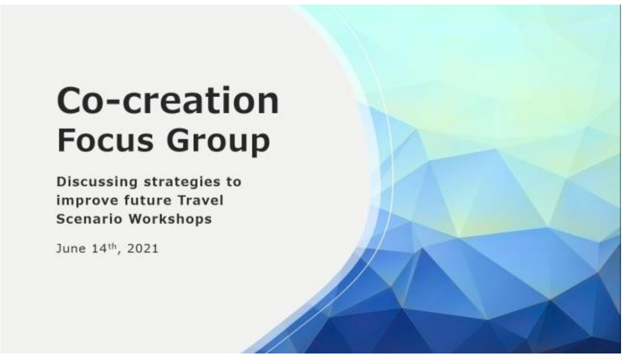<br><u>Overview</u><br>So this is a quick overview of what we will be doing today.<br>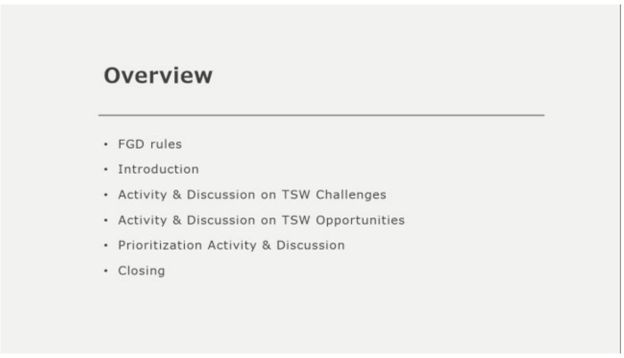<br>There will be some short breaks between the activities. Before continuing, if no one objects, I would like to start the recording<br><br><u>Rules/Ethics</u><br>To ensure a safe and open environment, we have some ground rules for this FGD.	To create a pleasant atmosphere, become acquainted with each other and introduce the topic of the FGD.

<table>
<tr>
<td></td>
<td></td>
<td>
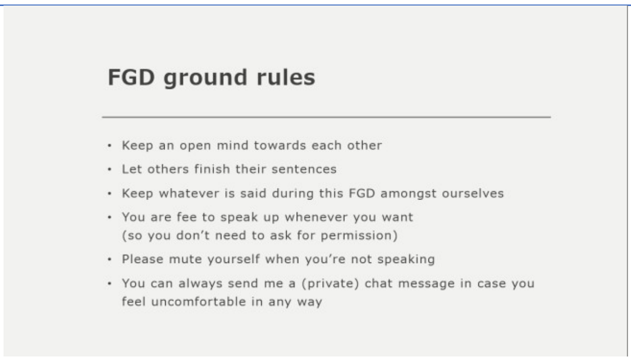

I hope that you all feel comfortable to speak freely, so we can have a productive and rich discussion in which all points of view are valid.

>> close PowerPoint presentation and put Zoom on gallery view <<

<u>Introductions</u>
So even though some of you already know each other, I would like to start with a short introduction round before we go into the activities. So please say something about yourself, and tell us what would be the first location that you would like to go/travel to when the COVID-19 situation allows us again. I will start and hand it over to the next person, please do so as well.

So I am Lotte, I am originally from a small town in the Netherlands and I am now finalizing my Research Masters in Global Health in Amsterdam. So next step for me is finding a job in Global Health. And if we are allowed to travel again I would really like to go hiking around the Lofoten islands in Norway. XXX, would you like to go next?
</td>
<td></td>
</tr>
<tr>
<td>Total:
30 min

Start time:
15.15
End time:
15.45</td>
<td>TSW Challenges

- Brainstorming on (missing) challenges
- Identify possible clusters based on underlying issues
- Discuss why these types of challenges persist
- Grade the identified challenges</td>
<td>
>> Share the 1st Jamboard link in the zoom-chat!<<

<u>Brainstorming on (missing) challenges</u>
Present (an read out to) them the identified challenges on Jamboard:
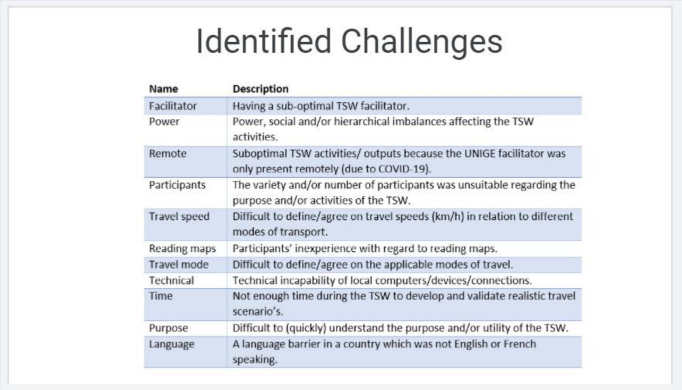
Identified Challenges
<table>
<tr><th>Name</th><th>Description</th></tr>
<tr><td>Facilitator</td><td>Having a sub-optimal TSW facilitator.</td></tr>
<tr><td>Power</td><td>Power, social and/or hierarchical imbalances affecting the TSW activities.</td></tr>
<tr><td>Remote</td><td>Suboptimal TSW activities/ outputs because the UNIGE facilitator was only present remotely (due to COVID-19).</td></tr>
<tr><td>Participants</td><td>The variety and/or number of participants was unsuitable regarding the purpose and/or activities of the TSW.</td></tr>
<tr><td>Travel speed</td><td>Difficult to define/agree on travel speeds (km/h) in relation to different modes of transport.</td></tr>
<tr><td>Reading maps</td><td>Participants' inexperience with regard to reading maps.</td></tr>
<tr><td>Travel mode</td><td>Difficult to define/agree on the applicable modes of travel.</td></tr>
<tr><td>Technical</td><td>Technical incapability of local computers/devices/connections.</td></tr>
<tr><td>Time</td><td>Not enough time during the TSW to develop and validate realistic travel scenario's.</td></tr>
<tr><td>Purpose</td><td>Difficult to (quickly) understand the purpose and/or utility of the TSW.</td></tr>
<tr><td>Language</td><td>A language barrier in a country which was not English or French speaking.</td></tr>
</table>
</td>
<td>To identify the challenges and possible (shared) underlying causes of the TSWs, and assess which of these challenges are most pressing (and should therefore be addressed).</td>
</tr>
</table>

<table>
<tr>
<td></td>
<td>- Show survey results on some challenges
- Possibly re-evaluate the indicated grades</td>
<td>

- Ask whether they recognize these challenges
- Ask whether there are challenges that they did not expected
  > maybe specifically ask a person for feedback/opinion
- Ask whether they think there are some missing challenges
  > add these to the next Jamboard page

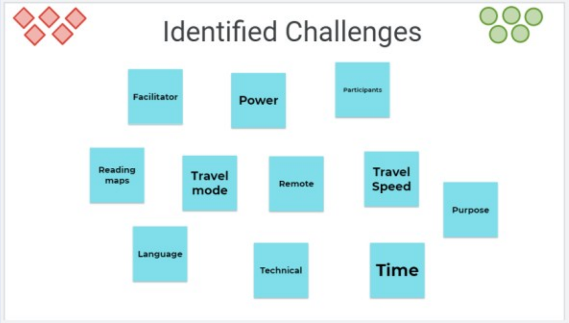

<u>Clustering</u>

- Discuss whether its possible to identify some clusters among the challenges
  > for example, are some challenges explained by the same underlying cause?
- Group those challenges together and name them (still on the same Jamboard page).
- Discuss why they think these challenges persist.

<u>Grading the challenges</u>

- To identify the most pressing challenges, ask each participant to assign one red diamond (= most pressing) and one green circle (=2nd most pressing) to the identified challenges or clusters.
- Discuss how they have come to their assessment.

<u>Survey results</u>

- Show some survey results on challenges in PowerPoint
  >> Open PowerPoint presentation again <<
  > purpose (example slide below)

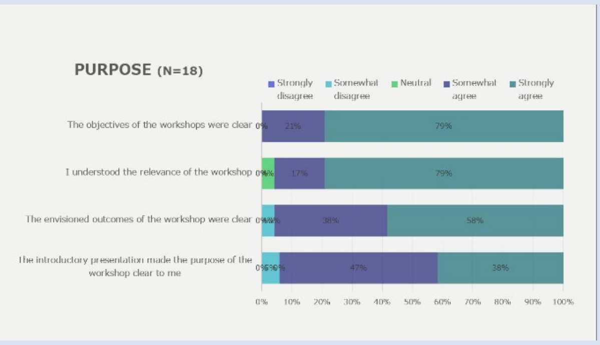

> power
</td>
<td></td>
</tr>
</table>

		> participants<br>> facilitators<br>> time<br>> reading maps<br>> travel speed<br>*>> close PowerPoint presentation <<*<br>• Ask if someone wants to change their assessment of most pressing challenge(s) on the Jamboard.<br>• Discuss why they did or did not change.	
BREAK (max. until 15.55) → prepare 2nd Jamboard page 3			
Total:<br>25 min<br><br>Start time:<br>15.55<br>End time:<br>16.20	**TSW opportunities**<br><br>- Brainstorming on (missing) opportunities in relation to the challenges<br>- Link the opportunities to the identified (clusters) of challenges<br>- Ranking strategies regarding travel speed.<br>- Discuss survey data on travel speed-, validation-, and preparation strategies.	<u>Brainstorming on (missing) opportunities:</u><br>So, how to address the identified (clusters) of challenges?<br>• I would like to start with a short individual brainstorm to identify possible opportunities<br>> you don't need to cover all discussed challenges (maybe focus on the most pressing ones)<br>> please keep it short<br>*>> start the MentiMeter & share screen <<*<br>• Please go to www.menti.com (it might be easier to do this on your smartphone) and enter the following code: **5339 2652**<br>> here you can shortly write some of your ideas, we will discuss them after.<br>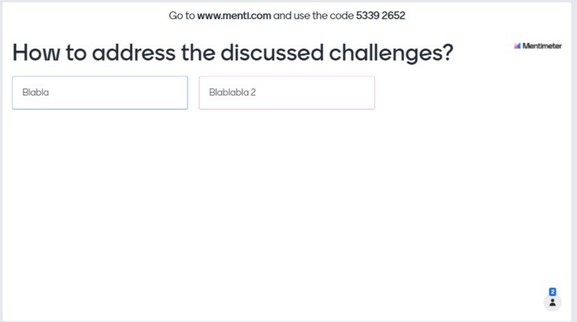<br>*>> make a screenshot and paste in Jamboard<<*<br>• Before discussing this in detail, I would like to ask you to go to another Jamboard (but please do not close the tab!)<br>*>> Share the 2nd Jamboard link in the zoom-chat!<<*<br>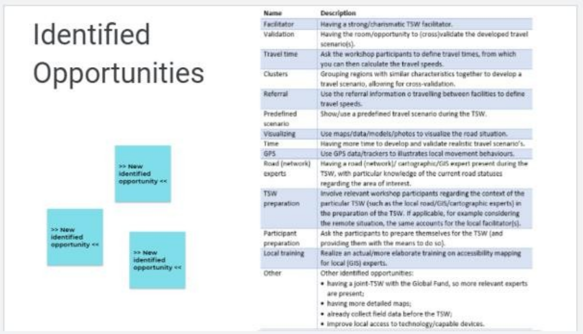	To identify opportunities in relation to the challenges and to discuss the relevance of some opportunities according to the survey data

- Here you see the identified opportunities from the interviews and surveys. I think a lot of what you suggested is covered by these opportunities, but please say so if you think yours is missing or if the description is not really correct.
- Discuss the overlap and differences
  >> adjust/add the possible new opportunities <<
  >> also add these to page 3 of the Jamboard <<

Linking the opportunities to the challenges

- On the third page of the Jamboard you find the challenge-areas (including pressing indicators?) and the identified opportunities.

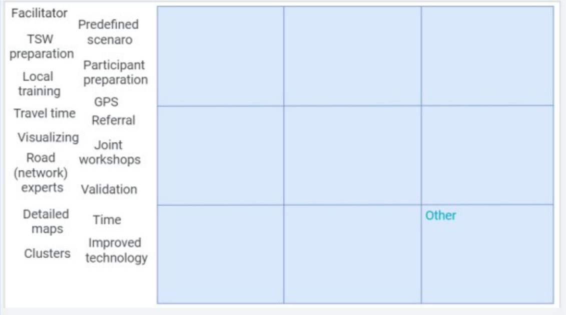

- I want to ask you to please move the opportunities to the challenge(s) that they address.
- Shortly discuss their activity and check whether there is consensus.

Strategies regarding travel speed

- Because assessing realistic travel speeds is found to be very challenging, I would first like to ask you individually to assess what strategy you think would be most effective to address this challenge. Therefore, we return to the MentiMeter
  >> go to next question in MentiMeter and share screen again <<

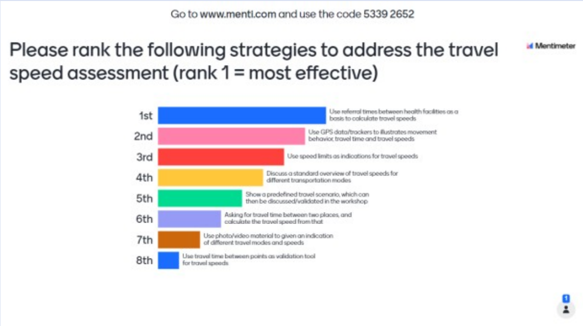

- I would like to ask you if you could rank these options, with number 1 being the most promising strategy
  >> Paste screenshot of menti outcome on page 4

		of the Jamboard <<<br>*>> Next to this screenshot, paste survey ranking from PPT in Jamboard page 4 <<*<br>• Discuss the differences.<br>• Find consensus.<br>• Discuss if an important one is missing, and if so, where it should be placed.<br><br><u>Regarding preparation & validation</u><br>• Before going into the last activity, I would like to show you the follow outcomes of the survey<br>*>> Open PowerPoint presentation again and share screen <<*<br>> regarding participant preparation<br>> regarding validation (clusters also showed to be used for validation in 50% of the times clusters were applied)	
BREAK (max. until 16.25) → Prepare 3th Jamboard page 1			
Total:<br>25 min<br><br>Start time:<br>16.25<br>End time:<br>16.50	**Prioritizing opportunities**<br><br>- Prioritize based on level of facilitation and feasibility of the opportunities/ strategies<br>- Discuss (extensively)<br>- Find consensus<br>- Individually identify most promising opportunity	*>> Share the 3rd Jamboard link in the zoom-chat!<<*<br><br><u>Prioritizing opportunities</u><br>• With the survey knowledge and all discussed before, I would like to ask you to prioritize the opportunities based on their level of facilitation (regarding how much they address the most pressing challenges) and their feasibility (regarding implementation in future workshops).<br>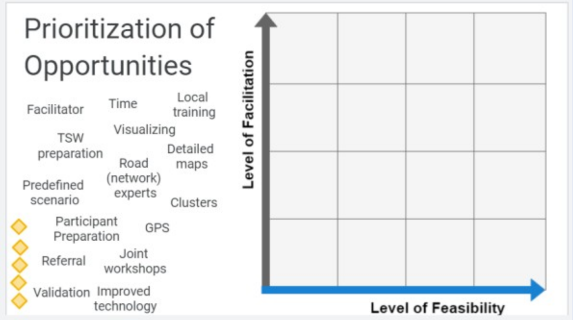<br>• Ask participants why they places them where they are.<br>• Discuss/find consensus on the overview.<br>• Let each participant place a golden diamond on the opportunity that most definitely should be implemented in future TSWs.	To prioritize the identified opportunities based on level of facilitation and feasibility
Total:<br>8 min<br><br>Start time:<br>16.50<br>End time:	**Brainstorm on guideline format**<br><br>- Discuss how to shape these ideas into a format	<u>Brainstorm on Guideline format</u><br>• Considering the implementation of these strategies/opportunities in future TSWs, as a last activity I would like to ask you how you think we can best communicate/document this. So how to	To record participant ideas for the format of the state-of-the-art guideline for travel scenario

16.58		best format these TSW improvement strategies for future application?<br>• Let them write sticky notes on the 2nd Jamboard page.<br>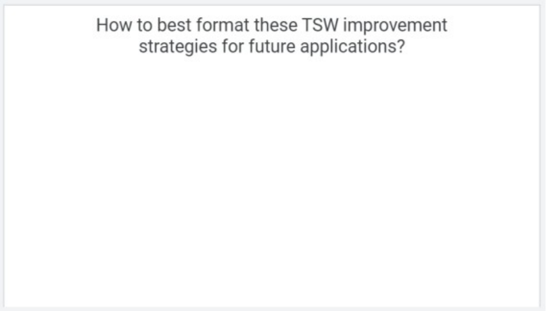<br><br>• If time allows, shortly discuss whatever is written and maybe try to find consensus on the best approach.	knowledge elicitation
Total:<br>2 min<br><br>Start time:<br>16.58<br>End time:<br>17..00	**Closing**<br><br>- Recap<br>- Thank the participants	<u>Closing:</u><br>So, we have come to the end of this very full FGD. To summarize, we have had a rich discussion on strategies to improve future travel scenario workshops. We identified challenges and opportunities, and we determined which opportunities should receive (the most) emphasis in a guideline to optimize and harmonize TSW activities and outputs.<br><br>Does anyone have any further questions or comments on this, before we wrap up?<br><br><u>Thank you:</u><br>I would like to thank you again for participating and I hope you also enjoyed this session. Your input and participation is very valuable for the research project. You can always contact me in case you have any questions or comments, and I hope you enjoy the rest of your day/evening.	To close the FGD nicely and thank the participants for their input and participation.
