# Supplementary material for: Travel scenario workshops for geographical accessibility modeling of health services: A transdisciplinary evaluation study
Source: Front Public Health. 2023 Jan 18;10:1051522. doi: 10.3389/fpubh.2022.1051522 (PMC9889992; doi:10.3389/fpubh.2022.1051522)
Supplement: Supplementary file 1 [file Data_Sheet_1.zip › Supplementary Information 4.PDF]

Initial Codebook

Code group	Code	Definition
Process	Countries	In what counties one was involved in the process.
	Experience	How one experiences the whole process.
	Perception	What one thinks of the process.
	Workshops	Explanation on the process-workshops besides the TSW.
Pre-TSW	Maps/model	Technical preparation.
	Meetings/liaison	Social preparation.
TSW	Activities/tools	The kind of activities and/or tools used during the TSWs.
	Countries	In what counties one was involved in the TSW + role.
	Experience	Things that happened during the workshops.
	Perception	What one thinks of the TSWs.
Post-TSW	Feedback	Feedback/discuss the travel scenario results and model outputs with the participants.
	Maps/model	Performing the accessibility analysis etc.
Facilitators	(Road)experts	Having road experts present during the workshop.
	Facilitator	Having a strong/charismatic facilitator.
	Feedback/time	Having enough time to make the travel scenarios and/or show the result of their travel scenario in the model.
	Visualizing	Using maps/data/models to visualize/explain.
	Other	…
Barriers	Participants	The (variety of) present stakeholders, as well as the number of present stakeholders.
	Power	Power imbalances or social factors.
	Reading maps	Inexperience with reading maps.
	Remote	Remote TSW/GeoHealth members cannot travel into the countries and be lively present at the TSW.
	Season	Influence of the dry/wet season on the travel scenario's
	Time	Not enough time to come to realistic travel scenario's.
	Travel speed	Defining travel speeds (in km/h)
	Other	…
Opportunities	Feedback	Discuss/Show the results of the formulated travel scenario(s).
	GPS	Use GPS trackers or systems.
	Prep-experts	Discussing objectives, relevance and details with facilitators, incorporating road experts.
	Prep-participants	Ask participants to prepare some things for the workshop.
	Referral	Use the referral information of travelling between health facilities.
	Travel time	Let the participants discuss time (between facilities, villages…) instead of speed.
	Other	…
Other	Demographics	Some personal information like age, occupation, career etc.

Other	Extra	Things for me to look into, or things to inquire with other interviewees.
	Networking	Info about possible other interviewees.

Final Codebook

Code group	Code	Definition
Process	Countries	In what counties one was involved in the process.
	Experience	How one experiences the whole process, including changes/developments and one's own role in it.
	Perception	What one thinks of the process.
	Workshops	Explanation on the process-workshops besides the TSW.
Pre-TSW	Maps/model	Technical preparation.
	Meetings/liaison	Social preparation.
TSW	Activities/tools	The kind of activities and/or tools used during the TSWs.
	Countries	In what counties one was involved in the TSW + role.
	Experience	Things that happened during the workshops.
	Learning	Learning experiences from the TSW (for participants/facilitators)
	Participants	Concerns the (kind of) stakeholders/experts present/ involved with the workshop and or results/outputs.
	Perception	What one thinks of the TSWs.
Post-TSW	Feedback	Feedback/discuss the travel scenario results and model outputs with the participants.
	Maps/model	Performing the accessibility analysis etc.
Facilitators	(Road)experts	Having road/cartographic/local GIS experts present during the workshop.
	Cluster	Grouping regions with similar characteristics together, so that the travel scenario automatically applies to the entire cluster (as opposed to a different scenario for every single region/district)
	Facilitator	Having a strong/charismatic facilitator.
	Feedback/time	Having enough time to make the travel scenarios and/or show the result of their travel scenario in the model.
	Participatory	Appreciated that the workshop was very participatory/active contribution
	Visualizing	Using maps/data/models to visualize/explain.
	Other	….
Barriers	Facilitator	Having a sub-optimal facilitator.
	Participants	The (variety of) present stakeholders, as well as the number of present stakeholders.

Code group	Code	Definition
Barriers	Power	Power imbalances or social factors, often in relation to reaching consensus.
	Purpose	Understanding the objective/utility of the workshop
	Reading maps	Inexperience with reading maps.
	Remote	Remote TSW/GeoHealth members cannot travel into the countries and be lively present at the TSW (as a result of COVID).
	Season	Influence of the dry/wet season on the travel scenario's
	Time	Not enough time to come to realistic travel scenario's.
	Travel speed	Defining travel speeds (in km/h) in relation to different modes of transport.
	Other	....
Opportunities	Feedback	Discuss/Show the results of the formulated travel scenario(s) for (cross) validation.
	GPS	Use GPS trackers or systems.
	Photo	Use of pictures to visualize the roads (considering nomenclature can be very different in every country.
	Predefined	Show/use predefined travel speeds/travel scenarios.
	Prep-experts	Discussing objectives, relevance and details with facilitators, incorporating road experts.
	Prep-participants	Ask participants to prepare some things for the workshop.
	Referral	Use the referral information of travelling between health facilities.
	Time	Having more time to do the workshop
	Travel time	Let the participants discuss time (between facilities, villages…) instead of speed.
	Other	…
Other	Demographics	Some personal information like age, occupation, career etc. (For In-depth interviews: their roles regarding the TSW are also coded with demographics)
	Extra	Things for me to look into, or things to inquire with other interviewees.
	Networking	Info about possible other interviewees.
