# Supplementary material for: Travel scenario workshops for geographical accessibility modeling of health services: A transdisciplinary evaluation study
Source: Front Public Health. 2023 Jan 18;10:1051522. doi: 10.3389/fpubh.2022.1051522 (PMC9889992; doi:10.3389/fpubh.2022.1051522)
Supplement: Supplementary file 1 [file Data_Sheet_1.zip › Supplementary Information 5.PDF]

Welcome and thank you so much for participating in this survey!

BACKGROUND INFORMATION:

In collaboration with the UNFPA and the University of Geneva, I (Lotte Molenaar) conduct an evaluation study of the travel scenario workshops, to help improve the UNFPA strategy towards increased access to quality Emergency Obstetrics and Newborn Care (EmONC).

Considering you participated in one or several of these workshops, your experiences and perceptions provide for valuable insights for this study. By means of collecting and comparing data from participants who were present in different workshops in different countries and/or settings, we aim to learn about the challenges and opportunities of the travel scenario workshops, so as to optimize the workshop set-up for future applications.

INFORMED CONSENT:

The survey will take roughly 12-18 minutes. At the start we will ask for some demographic information to help us understand the overall characteristics of the respondents. However, **all information and responses are completely anonymous**. Furthermore, you do not have to answer any questions that you do not want to answer, and you are always allowed to stop the survey at any given time.

This survey has no commercial purposes and there are no right or wrong answers! In case you have questions, you can always reach out to the principal researcher via: <mailadres>

Q1 I have read the text above and the information is clear to me. I freely and voluntarily choose to participate in this study.

○ Yes (1)

○ No (2)

End of Block: Introduction

Start of Block: Demographics

DEMOGRAPHIC INFORMATION:

Q2 How old are you?

____

Q3 What is your gender?

- ○ Male (1)
- ○ Female (2)
- ○ Non-binary / third gender (3)
- ○ Prefer not to say (4)

Q4 What is your first nationality?

____

Q5 In what country do you currently live?

____

Q7 What is the highest level of education that you have completed?

- No education (1)
- Primary education (2)
- Secondary education (high school) (3)
- Undergraduate education (college or university) (4)
- Postgraduate education (master or doctorate/PhD) (5)
- Prefer not to say (6)

Q8 Which of the following categories best describes your current employment status?

- Employed, working 1-20 hours per week (1)
- Employed, working 21-40 hours per week (2)
- Employed, working >40 hours per week (3)
- Unemployed, looking for work (4)
- Unemployed, not looking for work (5)
- Unable to work (6)
- Retired (7)
- Prefer not to say (8)

Q9 Which of the following categories best describes your current employment?

- Self-employed (1)
- Academic/university (2)
- National government/ministry (3)
- Regional government/office (4)
- Private hospital/health facility (5)
- Public hospital/health facility (6)
- NGO (7)
- UN organization (8)
- Work setting/employer is not applicable (9)
- Other (please specify) (10) ____
- Prefer not to say (11)

End of Block: Demographics

Start of Block: Memory refreshment & TSW participation

PARTICIPATION IN THE TRAVEL SCENARIO WORKSHOP(S):

The remaining of the survey will specifically focus on the travel scenario workshop(s) you participated in participated in. In case active memories of the workshop(s) have slightly slipped your mind, please have a look at the following overview:

<u>Goals of the workshop:</u>

By means of consensus among the participants, the workshop(s) were intended to:

1. Define the modes of transports, both on and off roads, used by pregnant women and (young) mothers.

2. Determine the average travel speeds on roads as well as off roads, considering pregnant women and (young) mothers.

3. Discuss the possible barriers for movement, and the role of seasonality.

<u>Workshop activities:</u>

This workshop was likely part of a larger regional EmONC workshop, or part of an AccessMod workshop. Although the exact course of activities might have deviated per workshop, the following activities were part of the workshop:

1. Workshop opening by the facilitator.

2. Presentation and explanation of the concept of accessibility modeling, AccessMod functionalities, and use cases in different countries, to demonstrate the relevance of the travel scenario workshop. To illustrate the story, some slides were used that may have looked like this:

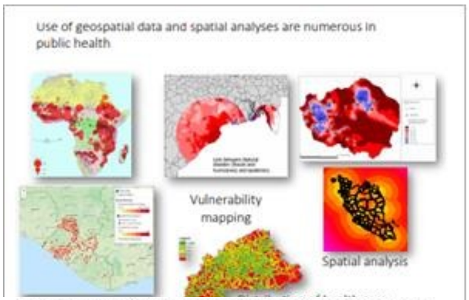

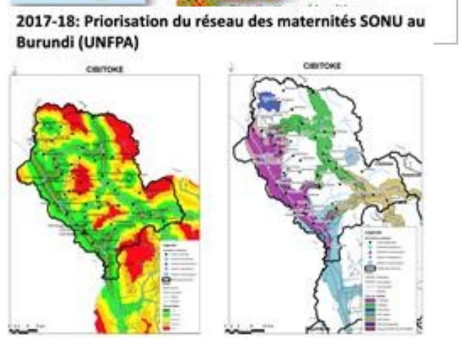

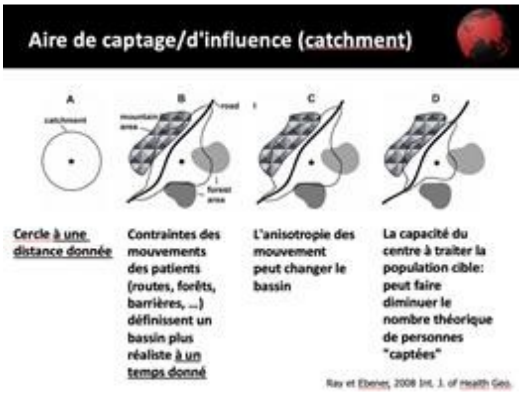

3. Working groups to define the modes of transport and travel speeds. The outcomes of the group work were (most likely) recorded in an Excel table which may have looked like this:

Roads	Conditions	Modes of transport	Frequency of use	Speed (km/h) dry season	Speed (km/h) wet season
Primary roads	Asphalted	Car			
		Motorcycle			
Secondary roads	Partially asphalted	Walking			
		Car			
		Motorcycle			
		Bicycle			
etc.					

4. Discussing the outcomes of the group work with all participants in a plenary session.

5. Closing statements by the facilitator.

6. The modes of transport and travel speeds were then used to model accessibility and produce maps for the process of prioritizing the EmONC network.

Q10 <u>Do the elements in this overview somewhat remind you of the travel scenario workshop(s) you participated in?</u>

○ Yes (1)

○ A bit (2)

○ No (3)

○ I don't know (4)

Page Break

PARTICIPATION IN THE TRAVEL SCENARIO WORKSHOP(S):

Q11 In what country(s) did you participate in a travel scenario workshop? *(multiple answers possible)*

- ○ Benin (2018) (1)
- ○ Burkina Faso (2020) (2)
- ○ Burundi (2017) (3)
- ○ Chad (2019) (4)
- ○ Democratic Republic of Congo (Maniema) (2020) (5)
- ○ Republic of Guinea (2018) (6)
- ○ Ivory Coast (2019) (7)
- ○ Madagascar (2018) (8)
- ○ Niger (2020) (9)
- ○ Republic of Congo (Sangha/Lekoumou provinces) (2019) (10)
- ○ Senegal (2018) (12)
- ○ Sudan (2018) (13)
- ○ Timor Leste (2020) (14)
- ○ Togo (2016) (15)
- ○ Other (please specify) (16) ____
- ○ None (17)

Q12 With which area(s) of expertise do you identify yourself most, considering your participation in the travel scenario workshop(s)? *(multiple answers possible)*

- ○ Cartographic expert (1)
- ○ Community/local health worker/expert (2)
- ○ (Delegate of the) Ministry of Health (3)
- ○ Geographic Information Systems (GIS) expert (4)
- ○ Maternal/reproductive health expert (5)
- ○ Medical practitioner/expert (6)
- ○ Midwifery expert (7)
- ○ Nursing expert (8)
- ○ Regional (health) director (9)
- ○ Hospital director (22)
- ○ Regional representative (10)
- ○ Road (network) expert (11)
- ○ UNFPA staff (12)
- ○ Other UN staff (13)
- ○ Staff of other international organizations (17)
- ○ NGO staff (18)
- ○ International consultant (19)
- ○ Other(s) (please specify) (20) ____
- ○ Prefer not to say (21)

Q13 Which of the following categories describes your role during the workshop best?

○ Workshop facilitator (1)

○ Supporting the workshop facilitator (please specify why/how) (2) ____

○ National representative participating in the group work to decide on the modes of travel and the travel scenario (3)

○ National representative participating in the group work to decide on the modes of travel and the travel scenario, and presenting the group work results in plenary (4)

○ Regional representative participating in the group work to decide on the modes of travel and the travel scenario (5)

○ Regional representative participating in the group work to decide on the modes of travel and the travel scenario, and presenting the group work results in plenary (6)

○ Other (please specify) (7) ____

○ Prefer not to say (8)

End of Block: Memory refreshment & TSW participation

Start of Block: Overall experiences & perceptions >> based on validated course surveys

OVERALL EXPERIENCES WITH-, AND PERCEPTIONS ON THE TRAVEL SCENARIO WORKSHOP(S):

Q14 Please indicate to what extent you (dis)agree with the following statements when you think about the travel scenario workshop(s) in which you participated

	Strongly disagree (1)	Somewhat disagree (2)	Neither agree nor disagree (3)	Somewhat agree (4)	Strongly agree (5)	I don't know (6)
The objectives of the workshop were clear to me (1)						
I understood the relevance of the workshop (2)						
The envisioned outcomes of the workshop were clear to me (3)						
Local examples were used to clarify the workshop materials (4)						
The workshop was interesting (5)						
I learned a lot during the workshop (6)						
The workshop materials were difficult to understand (7)						
I learned new things during the workshop that I now use in my personal life (8)						
I learned new things during the workshop that I now use in my professional life (9)						
I found this workshop challenging (10)						
The facilitator(s) were enthusiastic/charismatic (11)						
The facilitator(s) understood where extra support was needed (12)						
Discussion among the participants was promoted by the facilitator(s) (13)						

The communication between facilitator(s) and participants was good (14)	○	○	○	○	○	○
The workshop participants supported or helped each other when needed (15)	○	○	○	○	○	○
I was given the chance to actively participate in the workshop (16)	○	○	○	○	○	○
There was limited discussion between the participants of the workshop (17)	○	○	○	○	○	○
There was a friendly relationship between the workshop participants (18)	○	○	○	○	○	○
Participant contribution was somewhat equally distributed (19)	○	○	○	○	○	○
I sometimes felt unheard (20)	○	○	○	○	○	○
The workshop was well organized (21)	○	○	○	○	○	○
The facilitator(s) were sensitive to feedback (22)	○	○	○	○	○	○
The facilitator(s) were helpful when questions were asked (23)	○	○	○	○	○	○
The (local) cartographic experts were helpful when questions were asked (24)	○	○	○	○	○	○
The allocated time for the workshop was sufficient (25)	○	○	○	○	○	○

Page Break

OVERALL EXPERIENCES WITH-, AND PERCEPTIONS ON THE TRAVEL SCENARIO WORKSHOP(S):

Q15 Please assess the following aspects of the travel scenario workshop(s) on a scale from 0-10 *(where 10 represents perfection).*

	0 1 2 3 4 5 6 7 8 9 10
To what extent was the workshop a success?	
How would you assess your overall experience of the workshop?	
To what extent did you feel like you had the right expertise to contribute to the workshop?	

End of Block: Overall experiences & perceptions >> based on validated course surveys

---

Start of Block: Introduction/presentation

EXPERIENCES WITH-, AND PERCEPTIONS ON THE INTRODUCTORY PRESENTATION:

After the workshop opening, the travel scenario workshop started with a presentation. This presentation included information about: geospatial data, maps, examples of similar work in other countries, the AccessMod tool, accessibility to healthcare (for women in need of EmONC), catchment areas, modes and speeds of travel, seasons, and travel scenarios.

Q16 Please indicate to what extent you (dis)agree with the following statements, when you think about the introductory presentation.

	Strongly disagree (1)	Somewhat disagree (2)	Neither agree nor disagree (3)	Somewhat agree (4)	Strongly agree (5)	I don't know (6)
The presentation immediately made the purpose of the workshop clear to me (1)	○	○	○	○	○	○
The presentation helped me to see the relevance of the workshop (2)	○	○	○	○	○	○
The presentation made me (more) aware of the relationship between good roads and better access to emergency obstetrics and newborn care (3)	○	○	○	○	○	○
The presentation provided a sufficient introduction to AccessMod and its applications (4)	○	○	○	○	○	○
The terminology/language used in the presentation was easy to understand (5)	○	○	○	○	○	○
I thought it was difficult to read/understand the maps of my country/region (6)	○	○	○	○	○	○
I enjoyed seeing the cartographic visualization(s) of the country/region (7)	○	○	○	○	○	○
I would have appreciated it if the country/regional maps were more detailed, by means of indicating more familiar or recognizable places. (8)	○	○	○	○	○	○

The facilitator/presenter was well prepared (9)	o	o	o	o	o	o
The facilitator/presenter encouraged everyone to share their knowledge or ask questions (10)	o	o	o	o	o	o
I felt like the facilitator/presenter knew the reality of the field (11)	o	o	o	o	o	o
There was enough time/room to clarify everything before getting started with the group work (12)	o	o	o	o	o	o

---

Page Break

EXPERIENCES WITH-, AND PERCEPTIONS ON THE INTRODUCTORY PRESENTATION:

The introductory presentation was intended to prepare you for the group work to define the travel scenario(s) for your country/region. To develop these scenario(s), it was necessary to understand what was meant by-, and the relevance of- the item/terms listed below.

Q17 Please indicate per item **how** you came to understand what was exactly meant by it, and **who** helped you to get to this understanding?

	I came to my understanding.......					......helped me to really understand the meaning and relevance.			
	From the introdu during after tion (1)	From the questio ns and answers the present ation (2)	I alrea dy kne w meanin (3)	I did not really underst and the g and relevan ce (4)	Not applic able presenta ctory or the (5)	The facilitat or(s) (1)	The (local) road/GI S/ cartogra phic expert(s) (2)	Some one else presen t in the works hop (3)	Not applic able (4)
Types of roads (i.e. primary roads, tracks etc.) (1)	○	○	○	○	○	○	○	○	○
Road conditions (i.e. (not) asphalted, open environme nt etc.) (2)	○	○	○	○	○	○	○	○	○
Land cover (i.e. forest, sand/deser t etc.) (4)	○	○	○	○	○	○	○	○	○
Modes of transport/ means of transportat ion (i.e. car, walking etc.) (5)	○	○	○	○	○	○	○	○	○

Frequency of use of transportation modes (15)									
Movement barriers (i.e. rivers etc.) (6)									
Travel time (in minutes or hours) (7)									
Travel speed (in km/h) (8)									
Referral time (travel time between health facilities) (9)									
The relationships between road conditions and travel speeds (10)									
Impact of seasonality on travel speed (11)									
Catchment area(s) (population coverage per health facility) (12)									

Accessibility coverage (accessibility of (health) resources for population) (13)	○	○	○	○	○	○	○	○	○

Q18 In case the right materials and reimbursement for your time were offered to you, would you have prepared yourself for the workshop?

- ○ Yes, I would have liked to quite extensively familiarize myself with the goals, terms and materials before the workshop (1)
- ○ Yes, I would have liked to shortly familiarize myself with the goals, terms and materials before the workshop (2)
- ○ No, the introductory presentation was a suitable/sufficient method to become acquainted with the goals, terms and materials of the workshop (3)
- ○ No, because chances are high that I would not have found the time to prepare myself properly for the workshop (4)
- ○ I don't know (5)

Q19 Please assess on a scale from 0-10 how well the introductory presentation prepared you for the remaining of the workshop *(where 10 represents perfection)*.

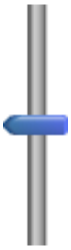

0 (0)
1 (1)
2 (2)
3 (3)
4 (4)
5 (5)
6 (6)
7 (7)
8 (8)
9 (9)
10 (10)

Q20 Do you have any suggestions of how the introductory presentation could be improved?

____

End of Block: Introduction/presentation

Start of Block: Groupwork

EXPERIENCES WITH-, AND PERCEPTIONS ON THE GROUP WORK:

After the introductory presentation you were subdivided into groups to define the modes of transport and travel speeds for the district/country/region you are most familiar with.

Q21 Please indicate to what extent you (dis)agree with the following statements, when you think about this group work practice.

	Strongly disagree (1)	Somewhat disagree (2)	Neither agree nor disagree (3)	Somewhat agree (4)	Strongly agree (5)	I don't know (6)
I enjoyed the group work (1)	○	○	○	○	○	○
I felt comfortable in the group I worked with (2)	○	○	○	○	○	○
There was enough time to develop the travel scenario(s) (3)	○	○	○	○	○	○
Naturally, our group was led by one of the participants (4)	○	○	○	○	○	○
I would have liked to fill in my own travel scenario table before discussing it in a group (5)	○	○	○	○	○	○
I feel like we had the right combination of experts in our group to develop a realistic travel scenario (6)	○	○	○	○	○	○
I think it is an added value to make different travel scenarios for the dry and the wet season (7)	○	○	○	○	○	○

I think it is an added value to make different travel scenarios for urban and rural areas (8)	○	○	○	○	○	○
Every group member contributed (equally) to the development of the travel scenario (9)	○	○	○	○	○	○
I was already familiar with (some of) the participants in my group (10)	○	○	○	○	○	○

Page Break

EXPERIENCES WITH-, AND PERCEPTIONS ON THE GROUP WORK:

Q22 How many people were (approximately) participating in your group?

____

Q23 According to you, what would be the ideal number of people in a group to work together on a travel scenario?

○ 2-5 (1)

○ 6-9 (2)

○ 10-13 (3)

○ >13 (4)

○ I don't know (5)

Q24 Please rank the following experts according to most relevant knowledge (1) to least relevant knowledge (9) regarding their ability to give input to define realistic travel scenarios. You can move the participants using drag and drop. *(If a particular expert was not present in your work group, please do still try to rank it based on what you think they could have added).*

_______GIS/Cartographic expert (1)

_______(Community) nurse/local health worker (2)

_______(Delegate of the) Ministry of Health (3)

_______Maternal/reproductive health expert (4)

_______Medical practitioner/expert (5)

_______Midwife/Midwifery expert (6)

_______Regional (health) director (7)

_______Road (network) expert (8)

_______UNFPA staff (9)

Page Break

EXPERIENCES WITH-, AND PERCEPTIONS ON THE GROUP WORK:

Q25 On a scale from 0-10 how difficult was it for you to assess travel speeds related to the different modes of transport, road conditions and considering different types of vegetation *(10 represents completely impossible).*

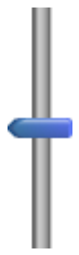

0 (0)
1 (1)
2 (2)
3 (3)
4 (4)
5 (5)
6 (6)
7 (7)
8 (8)
9 (9)
10 (10)

Q26 Please rank which of the following options would have been most helpful for you to best define the travel speeds related to the different modes of transport, road conditions and considering different types of vegetation *(1 represent the most promising option).*

_______Asking for the travel time between two places, and calculate the travel speed from that (1)

______Use the referral times between health facilities as a basis to calculate the travel speeds (2)

______Show a predefined travel scenario, which can then be discussed/validated based on the experience of the experts (3)

______Discuss a standard overview of average travel speeds for different modes of travel (4)

______Use photo/video material to given an indication of different travel speeds related to various modes of travel (5)

______Use GPS data/trackers to illustrates movement behavior, travel time and travel speeds (6)

______Use the travel time between certain points as validation tool for the travel speeds (7)

______Use speed limits at certain roads as indications/reference for travel speeds (8)

Q27 Overall, do you have any (other) suggestions of how the group work part of the travel scenario workshop could be improved?

____

End of Block: Groupwork

Start of Block: Plenary/discussion/after

EXPERIENCES WITH-, AND PERCEPTIONS ON THE PLENARY DISCUSSION AT THE END OF THE WORKSHOP:

As a last step of the travel scenario workshop, the defined travel scenarios where presented and/or discussed in plenary, with the aim to come to a consensus.

Q28 Please indicate to what extent you (dis)agree with the following statements, when you think about this plenary session at the end of the travel scenario workshop.

	Strongly disagree (1)	Somewhat disagree (2)	Neither agree nor disagree (3)	Somewhat agree (4)	Strongly agree (5)	I don't know (6)
There was enough time to have an all-encompassing plenary discussion about the developed travel scenarios (1)	o	o	o	o	o	o
The facilitator(s) led the plenary discussion well (2)	o	o	o	o	o	o
The participants kept engaged/motivated during the plenary discussion (3)	o	o	o	o	o	o
The (local) GIS/cartographic experts played a fundamental role during the plenary discussion (4)	o	o	o	o	o	o
During the plenary discussion, all different district/regional travel scenarios where presented to each other (5)	o	o	o	o	o	o
During the plenary discussion, the different district/regional travel scenarios were compared with each other (6)	o	o	o	o	o	o
During the plenary discussion, disagreements about travel scenarios or opinions emerged (7)	o	o	o	o	o	o
As a result of the plenary discussion, some travel scenarios were adapted (8)	o	o	o	o	o	o

The plenary discussion functioned as a validation/verification practice regarding the developed travel scenarios (9)	○	○	○	○	○	○
The plenary discussion functioned as an opportunity to share how everyone experienced the workshop (10)	○	○	○	○	○	○
The plenary discussion functioned as an opportunity to give feedback about the workshop (11)	○	○	○	○	○	○
The plenary discussion caused the travel scenarios to become more realistic (12)	○	○	○	○	○	○

Page Break

EXPERIENCES WITH-, AND PERCEPTIONS ON THE PLENARY DISCUSSION AT THE END OF THE WORKSHOP:

Q29 On a scale from 0-10, please assess to what extent you think your travel scenario represented the reality of the field *(where 10 represents perfection)*.

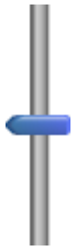

0 (0)
1 (1)
2 (2)
3 (3)
4 (4)
5 (5)
6 (6)
7 (7)
8 (8)
9 (9)
10 (10)

Q30 During the travel scenario workshop I attended, clusters of regions with similar geographical/travel characteristics were made, to limit the amount of different travel scenarios for one country.

○ No, this did not happen (1)

○ Yes we did, and in plenary we particularly discussed/compared the developed travel scenarios with the groups who were working on a region/district within the same cluster (2)

○ Yes we did, and we worked with the whole cluster on one travel scenario from the start (3)

○ Yes we did, but in plenary we did not specifically discuss/compare the developed travel scenarios with the groups who were working on a region/district within the same cluster (4)

○ I don't know (5)

Q31 In the following (workshop) days, were the maps suing your travel scenarios showed to-, or discussed with you? *(By this we mean the maps showing the accessibility analysis of health facilities for pregnant women and young mothers based on your table of modes and speeds of travel)*

○ No, but I would have liked to see it to verify if it represents my real life experience (1)

○ No, but I am very curious to see how our travel scenario looks on a map (2)

○ No, but I feel very confident about our developed travel scenario (3)

○ Yes, and based on the visualization we recognized some errors, so we made some changes in the scenario to make it more realistic (4)

○ Yes, and I feel the map was a good representation of reality (5)

○ Yes, but I found it hard to understand the visualization of the accessibility analysis (6)

○ Other (please specify) (7) ____

○ I don't know (8)

Q32 Overall, do you have any suggestions of how the plenary discussion at the end of thetravel scenario workshop could be improved?

____

End of Block: Plenary/discussion/after

Q33 Is there still something you would like to mention with regard to the travel scenarioworkshop(s) that you participated in?

____

End of Block: Finally
